# A Review on the Medicinal Plant* Dalbergia odorifera* Species: Phytochemistry and Biological Activity

**DOI:** 10.1155/2017/7142370

**Published:** 2017-12-04

**Authors:** Son Ninh The

**Affiliations:** Department of Bioactive Products, Institute of Natural Products Chemistry, Vietnam Academy of Science and Technology (VAST), 18 Hoang Quoc Viet, Cau Giay, Hanoi, Vietnam

## Abstract

The crucial medicinal plant* Dalbergia odorifera* T. Chen species belongs to genus* Dalbergia*, with interesting secondary metabolites, consisting of main classes of flavonoid, phenol, and sesquiterpene derivatives, as well as several arylbenzofurans, quinones, and fatty acids. Biological studies were carried out on extracts, fractions, and compounds from this species involved in cytotoxic assays; antibacterial, antioxidative, anti-inflammatory, antithrombotic, antiplatelet, antiosteosarcoma, antiosteoporosis, antiangiogenesis, and prostaglandin biosynthetic enzyme inhibition activities; vasorelaxant activities; alpha-glucosidase inhibitory activities; and many other effects. In terms of the valuable resources for natural new drugs development,* D. odorifera* species are widely used as medicinal drugs in many countries for treatment of cardiovascular diseases, cancer, diabetes, blood disorders, ischemia, swelling, necrosis, or rheumatic pain. Although natural products from this plant have been increasingly playing an important role in drug discovery programs, there is no supportive evidence to provide a general insight into phytochemical studies on* D. odorifera* species and biological activities of extracts, fractions, and isolated compounds. To a certain extent, this review deals with an overview of almost naturally occurring compounds from this species, along with extensive coverage of their biological evaluations.

## 1. Introduction

The medicinal plant* Dalbergia odorifera* T. Chen species, also called* Lignum Dalbergia odoriferae *[1], belongs to genus* Dalbergia*, family Fabaceae (Leguminosae) [2]. This plant has been widely distributed in the tropical regions of Central and South America, Africa, Madagascar, and East and Southern Asia [1, 3], especially in China [4].* D. odorifera* species, which has been known as “Jiangxiang” in Chinese, “Kangjinhyang” in Korean, and “Koshinko” in Japanese drugs, has been used in traditional medicine for the treatment of cardiovascular diseases, cancer, diabetes, blood disorders, ischemia, swelling, necrosis, rheumatic pain, and so on [5–7]. Particularly, from Chinese herbal preparations, heartwood was found and has been commonly employed as a part of commercial drug mixtures for cardiovascular treatments, including Qi-Shen-Yi-Qi decoction, Guanxin-Danshen pills, and Danshen injection [5, 6, 8–11]. As many other* Dalbergia* species, phytochemical investigations demonstrated the occurrence of the predominant flavonoid, phenol, and sesquiterpene derivatives in various parts of this plant, especially in terms of heartwood [12]. Furthermore, a number of bioactive reports on cytotoxic, antibacterial, antioxidative, anti-inflammatory, antithrombotic, antiosteosarcoma, antiosteoporosis, and vasorelaxant activities and alpha-glucosidase inhibitory activities indicate that both* D. odorifera *crude extracts and its secondary metabolites are valuable resources for new drugs development. However, no evidence was reported for the general view about this plant. In this review, we give an overview of the major chemical components and biological evaluations. This review would make a contribution to the understanding of the traditional values of* D. odorifera* and other related species, and it provides necessary guidelines for future researches.

## 2. Botany

According to database of The Plant List (http://www.theplantlist.org, 2017), the following acceptable name of* Dalbergia odorifera *T. Chen species is listed at a level of high confidence [13]. The medicinal plant* D. odorifera *species, also known as fragrant rosewood, is a semideciduous perennial tree [14], with morphological characteristics such as a height of 30–65 feet, oval leaves, and tiny yellow flowers [14]. Characteristic morphology has also been reported in the work of Hao and Wu (1993), based on the detailed description of the physical form and external structure made on stem parenchyma cells of a tropical deciduous tree of* D. odorifera* species [15]. As the outcomes displayed, in the secondary phloem of branchlet and trunk, the vacuole proteins were found in all of the parenchyma cells, except for companion cells. In addition, the proteins in the ray parenchyma and vasicentric parenchyma appeared in only the outer secondary xylem of the branchlet, but not in the trunk secondary xylem. The xylem vacuole proteins accumulated at the end of the growing period and disappeared after the first flush of growth in spring. The phloem vacuole proteins indicated seasonal variations, especially in the cells near the cambium. The fibrous structure of vacuole proteins was evidently found in the status of aggregation or in more or less even dispersion occurring in the large central vacuoles during both the growth and the dormant periods. Importantly, the nature of seasonal development in tropical trees might be different from that in temperate trees, in which a leguminous tree from the tropics of China such as* D. odorifera* species had the stem storage proteins in large central vacuoles, but the stem storage proteins of temperate trees appeared as small protein storage vacuoles or protein bodies, and the specific type of stem protein storage found in tropical plants might not be an accidental phenomenon [15].

The medicinal plant* D. odorifera* species has been shown as one of the most precious rosewoods in the world with diverse medicinal and high commercial values. For instance, its heartwood, named “Jiangxiang” in traditional Chinese medicine, was used in the Chinese Pharmacopoeia to treat cardiovascular diseases, cancer, diabetes, blood disorders, ischemia, swelling, necrosis, and rheumatic pain [6, 7]. As far as we know, the heartwoods provided a profitable resource of essential oils, which could be seen as a precious perfume fixative [1]. Apart from the important role in pharmaceutical industry, the heartwoods were famous for high-grade furniture and crafts, owing to their sweet fragrance, beautiful surface, and high density [2]. It is noticed that the wild plant* D. odorifera* species is threatened by habitat loss and overexploitation for timber usage [2, 16]. Therefore, the protection and growth of this one is an urgent task. Parallel with this, recently, the influence of geographic and temperature variations on* D. odorifera *seed germination (based on four geographic places: Ledong, Hainan; Pingxiang, Guangxi Zhuang Autonomous Region; Zhaoqing, Guangdong; and Longhai, Fujian, China) was reported in the work of Liu et al. (2017) [16]. The result revealed that the optimal germination temperature for seeds collected from Ledong and Pingxiang was 25°C, whereas that for seeds from the remaining two was 30°C. In another case, Lu et al. (2012) found out that the nodulating capacity to fix N_2_ from the atmosphere in* D. odorifera* species was a prerequisite for seedling establishment and growth, and we therefore need to identify the symbiosis relationship between strains of rhizobia and nodules of* D. odorifera* species [17]. Phylogenetic analysis of 16S rRNA gene and 16S–23S internal transcribed spacer (ITS) reckoned that these two bacterial strains, 8111 and 8201, were isolated from root nodules of an endemic woody legume in Southern China,* D. odorifera* species, which were closely related to* Burkholderia cepacia*. In the meantime, they were also similar in carbon source utilization using biology GN2 plate tests and their DNA G+C content was 65.8 and 65.5 mol%, respectively [17]. Two kinds of strains, 8111 and 8201, further provided high similarities with* B. cepacia *complex in the oxidation of almost all carbon sources, except for cellobiose, in comparison with* B. cepacia* and* B. pyrrocinia *by the oxidation of cellobiose and xylitol and with* B. vietnamiensis* by the oxidation of adonitol and cellobiose [17]. Additionally, plant biomass and N content showed that active N_2_ fixation occurred in nodules after inoculation with these two* Burkholderia *strains, as compared to negative control seedlings of* D. odorifera* species [17]. In conclusion,* Burkholderia* strains 8111 and 8201 can play positive roles in forming functional nodules of legume species* D. odorifera* [17].

Endophytic fungi or endophytes, existing widely inside the healthy tissues of plants, might significantly influence the formation of metabolic products and the quality and quantity of natural products derived from medicinal plants [49]. The relationship between diverse fungi and partial irregular heartwood of Guangdong, China,* D. odorifera* species, was reported by Sun et al. (2015); first, only two fungi were isolated from 160 white healthy wood tissues, approximately seven years old, which were affiliated to species of Bionectriaceae. On the contrary, 85 fungi were identified from purple or purple-brown wounded wood tissues, approximately seven years old, and belonged to 12 species [2]. Second, molecular identification and phylogenetic analysis showed that the isolated fungi performed seven distinct clades with a majority of the bootstrap values well above 90%, including* Fusarium *sp., Bionectriaceae, Pleosporales,* Phomopsis *sp.,* Exophiala jeanselmei*,* Auricularia polytricha*, and* Oudemansiella *sp. For example, the ITS sequence from the isolated code 12120 from wounded wood was identified as* Phomopsis *sp. and was clustered by 98% bootstrap support with* Phomopsis *sp. DQ780429 or with the isolated code 12201 derived from white healthy wood, exerting a strongly supported clade with* Bionectriaceae* sp. EF672316, especially three isolates 12119, 12130, and 12131 that were closely related by a 92% bootstrap value, which clustered strongly with the reference sequences of* Fusarium *sp. in GenBank. Third, extensive research and overall analyses of the endophytic isolation frequency exposed twelve fungal species in the purple-brown wounded wood in which the total colonization frequency was 53.125%, belonging to eight genera or families:* Eutypa*,* Fusarium*,* Phomopsis*,* Oudemansiella*,* Eutypella*,* Auricularia*,* Pleoporales* sp., and* Exophiala*, in which* Eutypa* sp. (12123) was the most frequent with 21.25%, whereas only* Bionectriaceae* sp. (1.25%) was found in the healthy white wood. Finally, anatomical analysis suggested that some fungal hyphae appeared in the vessels of purple-brown wounded wood, whereas this one was not found in the vessel of healthy white wood [2].

## 3. Chemistry

**Figure 1:**
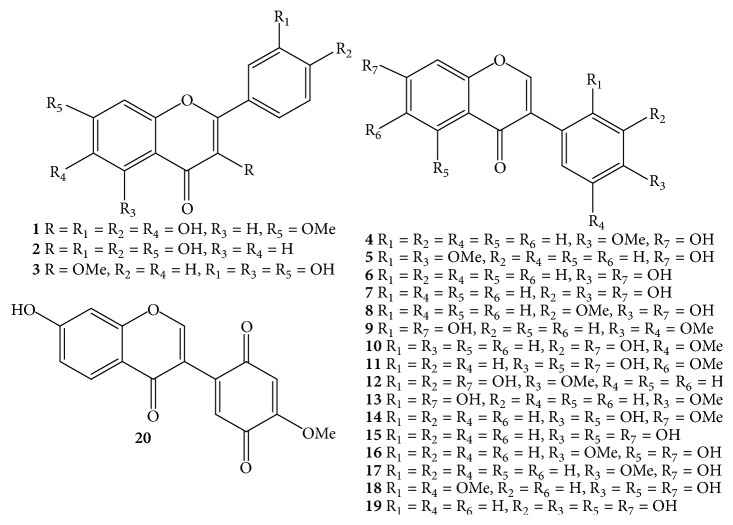
Flavones (**1**–**3**) and isoflavones (**4**–**20**) from* Dalbergia odorifera *species.

**Figure 2:**
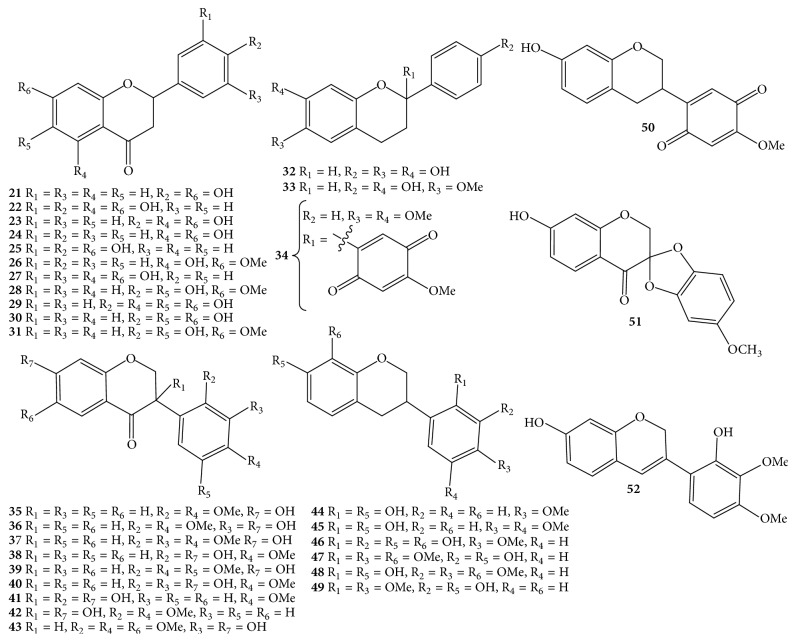
Flavanones (**21**–**31**) and flavans (**32**–**34**), isoflavanones (**35**–**43**), and isoflavans (**44**–**52**) from* Dalbergia odorifera *species.

**Figure 3:**
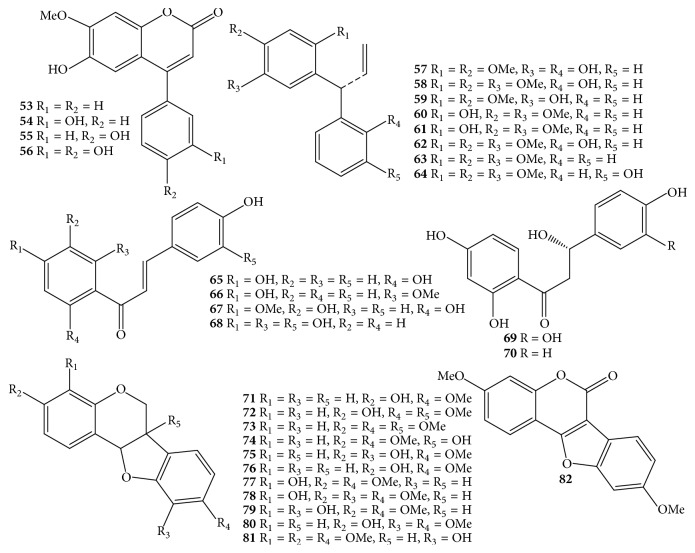
Neoflavones** (53**–**64)**, chalcones** (65**–**70)**, and pterocarpans** (71**–**82)** from* Dalbergia odorifera *species.

**Figure 4:**
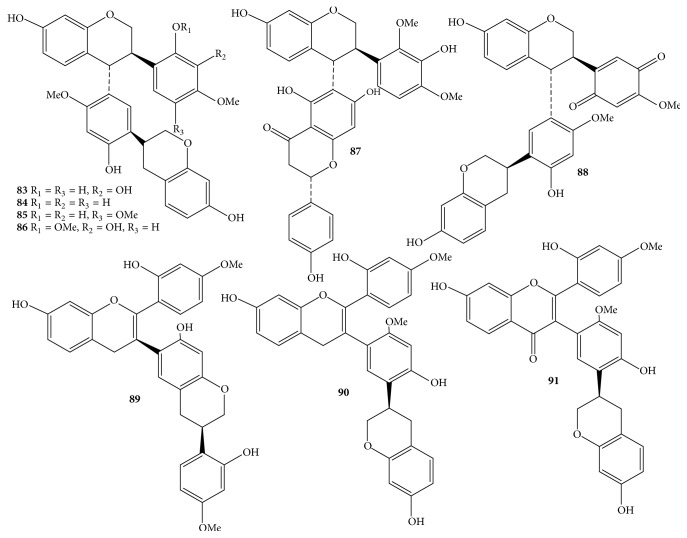
Bisflavonoids** (83**–**91)** from* Dalbergia odorifera *species.

**Figure 5:**
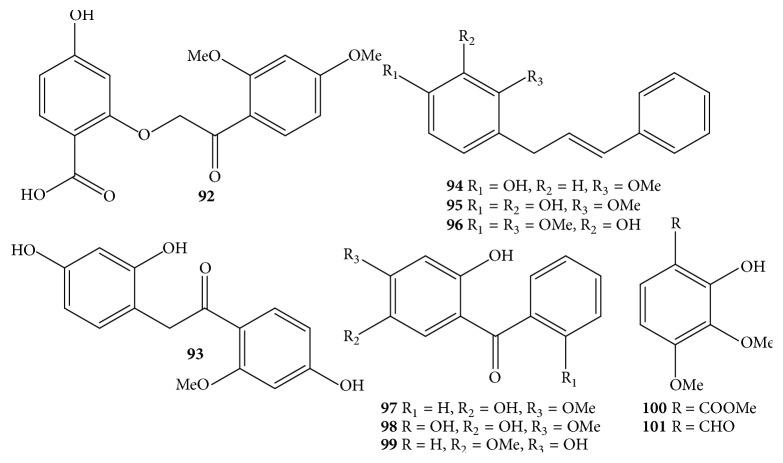
Phenols** (92**–**101)** from* Dalbergia odorifera *species.

**Figure 6:**
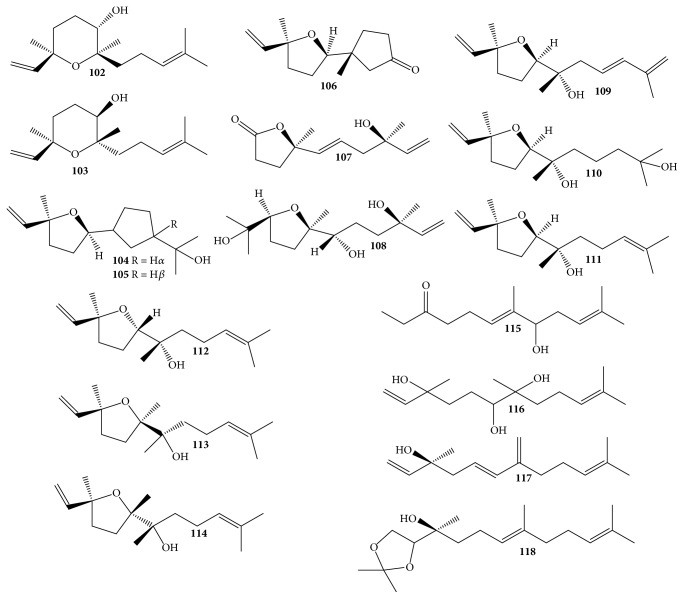
Sesquiterpenes** (102**–**118)** from* Dalbergia odorifera *species.

**Figure 7:**
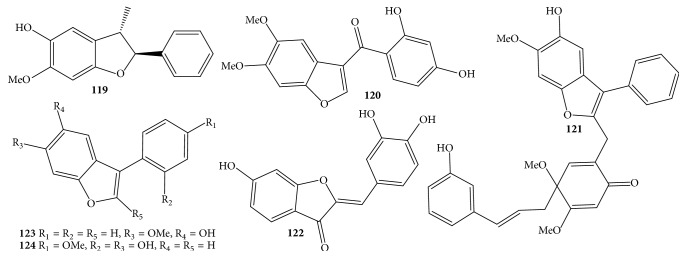
Arylbenzofurans** (119**–**124)** from* Dalbergia odorifera *species.

**Figure 8:**
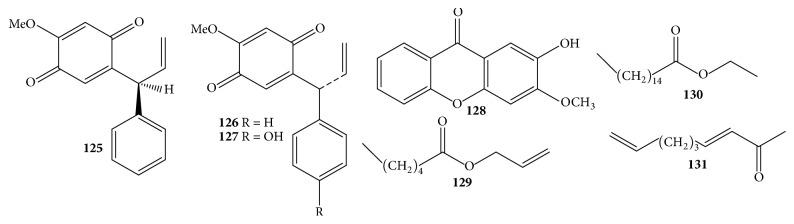
Quinones** (125**–**127)** and other components** (128**–**131)** from* Dalbergia odorifera *species.

Due to the economic value of* D. odorifera *species, it received much more attention from phytochemists. Nowadays, the processes of isolation, purification, and structure elucidation of interesting secondary metabolites are facilitated by continual development of chromatographic techniques such as thin-layer chromatography (TLC), column chromatography (CC), gas chromatography (GC), high-performance liquid chromatography (HPLC), ultraperformance liquid chromatography (UPLC), and spectroscopic analyses, for instance, nuclear magnetic resonance (NMR) and mass spectrum (MS). Apart from the chemical constituents only detected by HPLC and GC-MS, components of* D. odorifera* species are classified into a wide range of compounds, including flavonoids** 1**–**91** (Table 1 and Figures 1–4), phenols** 92**–**101** (Table 1 and Figure 5), sesquiterpenes** 102**–**118** (Table 1 and Figure 6), arylbenzofurans** 119**–**124** (Table 1 and Figure 7), quinones** 125**–**127**, and several other components** 128**–**131** (Table 1 and Figure 8).

### 3.1. Flavonoids

Flavonoid derivatives** 1**–**91** were obtained as major components from either* D. odorifera *or other species of the genus* Dalbergia *[12]. In general, phytochemical studies on* D. odorifera *species phytochemistry showed that most of the naturally occurring mono- and bisflavonoids occurred as free forms, and their glycosyl derivatives were seldom found. As shown in Table 1 and Figures 1–4, flavonoids** 1**–**91** can be divided into six different groups: flavones** 1–3**, and isoflavones** 4–20**; flavanones** 21–31**, flavans** 32–34**, isoflavanones** 35–43**, and isoflavans** 44–52**; neoflavones** 53–64**; chalcones** 65–70**; pterocarpans** 71–82**; bisflavonoids** 83–91**. The chemical index showed that most of the isolated flavonoids were isolated from heartwood. In addition, an isoflavone formononetin** (4)**, two flavanones (2*S*)-liquiritigenin** (21)** and (2*S*)-pinocembrin** (24)**, an isoflavanone (3*R*)-sativanone** (35)**, a chalcone isoliquiritigenin** (65)**, and a pterocarpan medicarpin** (71)** were found more often in the heartwood of* D. odorifera *species (Table 1). Of these bisflavonoids, chromatographic isolation of heartwood methanol extract provided nine new compounds** 83**–**91**, which belonged to dimeric isoflavanones and isoflavanones [20, 40]. In contrast to the conclusion of Saha et al. (2013), dimeric flavonoids were found only from* D. nitidula *and* D. monetaria* species [12].

### 3.2. Phenols

In the same manner as the class of flavonoids, phenolics are displayed as renowned components of the genus* Dalbergia *[12]. Phenolic compounds** 92**–**101** from* D. odorifera* species consist of simple structures (Table 1 and Figure 5) [7, 18, 19, 21–23, 28, 36]. Two new compounds named 2-(2-(2,4-dimethoxyphenyl)-2-oxoethoxy)-4-hydrobenzoic acid** (92)** and 2-(2,4-dihydroxyphenyl)-1-(4-hydroxy-2-methoxyphenyl)ethanone** (93)** were isolated from 95% ethanol extract of heartwood [7]. Meantime, the known ones** 94**–**99** were phenolic derivatives with skeleton of cinnamyl phenols or benzophenones separated from heartwood, root heartwood, or root [18, 19, 21–23, 28, 36].

### 3.3. Sesquiterpenes

Phytochemical and NMR structural elucidations also reported the existence of essential oils, which were sesquiterpenes** 102**–**118** (Table 1 and Figure 6) [41, 42]. Significantly, most of these compounds were identified as sesquiterpene alcohols. Seven new natural sesquiterpene alcohols, that is,* rel*-(3*R*,6*R*,7*S*)-3,7,11-trimethyl-3,7-epoxy-1,10-dodecadien-6-ol** (103)**,* rel*-(3*S*,6*R*,7*S*,10*S*)-2,6,10-trimethyl-3,6,7,10-diepoxy-2-dodecen-11-ol** (104)**,* rel*-(3*S*,6*R*,7*S*,9*E*)-3,7,11-trimethyl-3,6-epoxy-1,9,11-dodecatrien-7-ol** (109)**,* rel*-(3*S*,6*R*,7*S*)-3,7,11-trimethyl-3,6-epoxy-1-dodecen-7,11-diol** (110)**, (3*S*,6*R*,7*R*)-3,7,11-trimethyl-3,6-epoxy-1,10-dodecadien-7-ol** (113)**, (3*S*,6*S*,7*R*)-3,7,11-trimethyl-3,6-epoxy-1,10-dodecadien-7-ol** (114)**, and (3*S*,5*E*)-3,11-dimethyl-7-methylenedodaca-1,5,10-trien-3-ol** (117)**, were reported to be isolated from the* D. odorifera* heartwood [41, 42]. Previous phytochemical studies suggested that* trans*-nerolidol predominated in the essential oils of heartwood [1, 41], while chemical components** 102**–**118** were very closely related to this one in the biosynthetic pathways and structural manners (Figure 6) [41].

### 3.4. Arylbenzofurans

Regarding benzofuran derivative compounds, this typical class was not well known for the genus* Dalbergia *[12]; however, the naturally occurring benzofurans** 119**–**124** were available in* D. odorifera *species [4, 7, 28, 34, 35, 39, 40]. The most striking feature of these heterocyclic chemical compounds was aryl units directly or indirectly substituted at carbon C-2 or C-3 in the furan ring, while fused benzene rings were characterized by typical signals of an ABX spin system or typical signals of singlet protons H-4 and H-7 and methoxylation and/or hydroxylation for carbons C-5 and C-6 (Table 1 and Figure 7). Unfortunately, a new arylbenzofuran named 2′,6-dihydroxy-4′-methoxy-2-arylbenzofuran** (124)** might have the same structure as a known compound 6-hydroxy-2-(2-hydroxy-4-methoxyphenyl)benzofuran [39, 40].

### 3.5. Quinones and Other Components

A few quinones in the heartwood could have been observed with the biotransformation of phenyl units in compounds** 57**–**64** into quinonyl units in compounds** 125**–**12**7 (Table 1 and Figure 8) [18, 28, 36]. This phenomenon was also detected in flavonoids, for instance, compound** 50**. Finally, minor components** 128**–**131**, including 2-methoxy-3-hydroxyxanthone** (128)**, hexanoic acid, 2-propenyl ester** (129)**, hexadecanoic acid, ethyl ester** (130)**, and 3,8-nonadien-2-one** (131)**, were reported to exist in the heartwood and root so far [19, 24].

### 3.6. Qualitative and Quantitative Analyses

Additional information was provided about phytochemical investigations of* D. odorifera *species, which were further observed by HPLC, UPLC, GC-MS, and other modern technical analyses. Extensive research results using HPLC-UV, HPLC-MS, HPLC-DAD-ESI-MS, and LC-MS/MS experiments were published by Liu et al. (2005), Zhao et al. (2013), Ham et al. (2015), Fan et al. (2017), and Choi et al. (2017), in which a total of thirty-nine compounds** 4**–**7**,** 9**-**10**,** 12**,** 14**,** 21**,** 23**-**24**,** 33**,** 35**–**38**,** 41**,** 50**,** 53**–**57**,** 65**-**66**,** 68**,** 71**,** 80**,** 99**,** 126**-**127**, 4′-*O*-methyl-melanettin, 5,7-dihydroxy-2′,4′-dimethoxyisoflavone, 7,2′,3′-trihydroxy-4′-methoxyisoflavone, 2′,4′,5-trihydroxy-7-methoxyisoflavone, butin, alpinetin, homoferreirin, and piperidine, as well as four unknown components [component 1: *t*_*R*_ 23.4 min, *λ* 295 nm,* m/z* 341.2 [M+Na]^+^, 357.2 [M+K]^+^, 317.0 [M−H]^−^; component 2: *t*_*R*_ 40.4 min,* m/z* 339.2 [M+Na]^+^, 317.2 [M+H]^+^, 355.1 [M+K]^+^, 315.0 [M−H]^−^; component 3: *t*_*R*_ 86.8 min, *λ* 228 and 280 nm,* m/z* 369.2 [M+Na]^+^, 385.1 [M+K]^+^, 345.1 [M−H]^−^; and component 4: *t*_*R*_ 94.3 min, *λ* 260 nm,* m/z* 395.0 [M−H]^−^], were found in* D. odorifera* heartwood [29, 50–53]. By far, utilizing HPLC-UV/HPLC-MS techniques, eighteen flavonoids appeared in a rat serum sample collected at 30 min after oral administration of 75% heartwood ethanol extract, comprising compounds** 4**-**5**,** 7**,** 9**,** 12**,** 21**,** 36**–**38**,** 41**,** 53**–**56**,** 65**,** 68**,** 127**, and butin [54]. In other cases, the traditional Chinese medicine “Jiangxiang” was simultaneously analyzed by an offline 2D RPLC/RPLC system incorporating a *β*-CD based column and an Acuity UPLC BEH C18 column, in which 19 compounds were tentatively identified, comprising compounds** 71**,** 78**,** 87**,** 89**,** 97**, luteolin, melilotocarpan, 7,5,3′,4′-tetrahydroxyflavanone, three unknown trihydroxy-trimethoxyisoflavanones [the same molecular formula C_16_H_13_O_6_;* m/z* 301.0766 [M−H]^−^ and *t*_*R*_ 4.83 min;* m/z* 301.0766 [M−H]^−^ and *t*_*R*_ 4.59 min;* m/z* 301.0702 [M−H]^−^ and *t*_*R*_ 6.34 min], two unknown dihydroxy-monomethoxyisoflavanones [the same molecular formula C_16_H_15_O_4_;* m/z* 271.0895 [M−H]^−^ and retention time *t*_*R*_ 0.48 min;* m/z* 271.1017 [M−H]^−^ and *t*_*R*_ 1.07 min], two unknown trihydroxyflavanones [the same molecular formula C_15_H_11_O_5_ and* m/z* 271.0652 [M−H]^−^; *t*_*R*_ 3.30 and 6.62 min, resp.], three unknown dihydroxy-trimethoxyisoflavanones [the same molecular formula C_18_H_19_O_6_ and* m/z* 331.1109 [M−H]^−^; *t*_*R*_ 0.46, 0.76, and 1.25 min, resp.], and one unknown trihydroxy-dimethoxyisoflavanone [C_17_H_15_O_6_;* m/z* 331.0908 [M−H]^−^; *t*_*R*_ 5.94 min] [55]. Meanwhile, ten flavonoids** 4**,** 13**,** 21**,** 23**–**25**,** 35**-**36**,** 38**, and** 41** were isolated and identified after optimizing the separation and collection parameters from* D. odorifera* species using 2D Prep HPLC method with Click Oligo (ethylene glycol) and C18 column [27]. In the qualitative UPLC analysis for* D. odorifera* species in Hai'an, China, formononetin** (4)**, genistein** (15)**, and their glycosides (genistin and formononetin-8-C-apiosyl(1-6)-glucoside) were detected, and the authors suggested that matrix solid-phase dispersion using titania column (MSPD) was used to elute high concentration flavonoid aglycones first with 90% acetonitrile and 10% water containing 100 mM ammonium acetate buffer, followed by eluting trace flavonoid glycosides with 20% acetonitrile and 80% water containing 1% trifluoroacetate [30]. On the one hand, using HPLC-UV for qualitative analysis and deep eutectic solvent-based negative pressure cavitation assisted extraction (DES-NPCE) followed by macroporous resin column chromatography for quantitative analysis, the maximum extraction yields of four main isoflavonoids** 11** and** 14**–**16** accounted for 1.204, 1.057, 0.911, and 2.448 mg/g dry weight* D. odorifera *leaves, respectively, on which three effective factors for extraction were negative pressure −0.07 MPa, temperature 45°C, and concentration of water 26% [31]. On the other hand, following the efficient microwave-assisted aqueous two-phase extraction (MA-ATPE) technique and optimized conditions, for instance, dipotassium hydrogen phosphate salt concentration 20%, absolute alcohol concentration 25%, and extraction temperature 45.5°C, the extraction yields of genistein** (15)** and biochanin A** (16)** reached 1.023 and 2.012 mg/g dry material (DM), and the content in extracts possessed 12.966 and 25.526 mg/g extract, respectively, in which the MA-ATPE method exhibited about 2-3-fold higher value than those of microwave-assisted extraction (MAE) [the extraction yields of 0.899 and 1.915 mg/g DM; content in extracts of 5.212 and 11.101 mg/g extract, resp.] and heat reflux extraction (HRE) [the extraction yields of 0.924 and 1.715 mg/g DM; content in extracts of 4.897 and 9.086 mg/g extract, resp.] [14]. Similarly, based on optimal conditions including three extraction cycles, time 20 min, negative pressure −0.05 MPa, ethanol concentration 66%, and liquid/solid ratio 24 : 1 mL/g, NPCE extraction method showed that the extraction yields of compounds** 15-16** were 1.579 and 0.935 mg/g, respectively, when compared with 1.212 and 0.941 mg/g for HRE and 1.402 and 0.914 mg/g for ultrasound-assisted extraction (USE) methods, respectively [56]. Furthermore, using the combination of AL-2 macroporous resin and flash chromatography conditions [silica gel;* n*-hexane : ethyl acetate; sample : silica gel ratio 1.3 : 40; and flow rate: 50 mL/min], the content of compounds** 15-16** in the enriched product of leaves reached 27.20% and 6.79% [32].

The ethyl acetate extract (EE) of* D. odorifera *dried powdered seeds provided the highest total phenolic content at 563.2 ± 11.3 mg gallic acid equivalent/g extract although the extract yield was only 1.1% and was estimated to be approximately 3.3-, 4.1-, and 4.1-fold higher than those of* n*-butanol extract (BE), petroleum extract (PE), and water extract (WE), respectively [57]. Analogously, the EE extract also achieved the highest flavonoid content at 350.3 ± 3.1 mg rutin equivalent/g extract, establishing nearly 4.3-, 3.4-, and 3.0-fold higher values than those of BE (the extract yield was 7.0%), PE (1.1%), and WE (15.6%), respectively [57].

Taking essential oils into consideration, seeds of* D. odorifera* species grown in Hainan, China, produced volatile and liquid aroma compounds [1]. As far as the article reported, the chemical compositions were firstly obtained by simultaneous distillation and extraction (SDE) and analyzed by GC-MS, in which thirty-one compounds representing 93.8% of the essential oil were found with main components such as P,P,P-triphenyl phosphine imide (35.3%), bis(1-methylethyl)peroxide (16.4%), 1-methyl-1H-pyrrole (5.2%), 3,3,6-trimethyl-1,5-heptadien-4-one (4.7%), 1H-pyrrole (3.9%), 4-ethenyl-2-methoxy-phenol (3.9%), 2-*β*-pinene (3.5%), 3-(1-methylethyl)phenol (2.0%), formic acid* n*-pentyl ester (2.0%), glycidol (1.7%), and phenol (1.6%). Particularly, there was quite a difference in chemical oils among seeds, leaves, and heartwood, where major components in the oil of seeds extract were rarely found in the leaves and heartwood [1]. In other situations, in order to evaluate nutritional values of* D. odorifera* species in Hainan, China, the physical and chemical properties of seed oils were also obtained using 50% methanol at 50°C and 2 h and further extracting with petroleum ether at 30–60°C and 8 h in the Soxhlet extractor [58]. The results in describing the presence of major fatty acids linoleic acid (60.03%), oleic acid (17.48%), and palmitic acid (16.72%), along with the total tocopherol, total phenol, and *β*-carotene, were 511.9, 351.1, and 62.2 mg/kg oil, respectively. In addition, protein, carbohydrate, moisture, ash, and total phenolic contents were also found to be 12.96, 26.86, 42.58, 13.70, 3.90, and 5.55%, respectively, whereas physical properties such as free fatty acids, iodine number, peroxide value, saponification number, and unsaponifiable matter were 1.66%, 106.53 g/100 g, 5.07 meq O_2_/Kg, 196.78 mg KOH/g, and 1.70%, respectively.

## 4. Biological Activities

### 4.1. Cytotoxic Activities

The cytotoxic activity of chemical constituents of* D. odorifera* species is related to their structure and the organisms that they affect. Phytochemical investigation from the heartwood of* D. odorifera* species led to the isolation and structure elucidation of nine new compounds** 34**,** 43**,** 51**,** 72-73**,** 92-93**, and** 120-121**, along with five known ones** 74**–**77** and** 79**, which were all tested against human chronic myelogenous leukemia cell line** (**K562), human gastric carcinoma cell line (SGC-7901), and human hepatocellular carcinoma cell line (BEL-7402) [7, 35]. However, the inactive results had been received for all tested compounds except for only two components** 72** and** 121**, in which (6a*R*,11a*R*)-6a,9-dimethoxy-3-hydroxypterocarpan** (72)** showed the IC_50_ values of 15.9 and 12.7 *μ*M against SGC-7901 and BEL-7402 cell lines, respectively; meantime, phenylbenzofuran I** (121)** gave the IC_50_ value of 33.5 *μ*M against BEL-7402 cell line, when compared to the IC_50_ values of 1.87 and 7.38 *μ*M against SGC-7901 and BEL-7402 cell lines, respectively, for positive control paclitaxel [7, 35].

To further investigate* in vitro* cytotoxicity assays, Choi et al.** (**2009) reported the inhibitory effects of nine compounds** 4**,** 11**,** 21**,** 45**,** 49-50**,** 71**,** 95**, and** 101** on the proliferation of four human tumor cell lines, that is, human uterine carcinoma cell line (MES-SA), multidrug-resistant subline of MES-SA (MES-SA/DX5), human colorectal adenocarcinoma cell line (HCT-15), and multidrug-resistant subline of HCT15 (HCT15/CL02) [23]. Among them, two compounds, a pterocarpan medicarpin** (71)** and a phenolic compound hydroxyobtustyrene** (95)**, established the significant ED_50_ values with ranges of 5.7–7.3 and 5.1–6.8 *μ*M, respectively, whereas the remaining seven indicated moderate or inactive cytotoxicities, while those for positive control doxorubicin were found in a range of 0.0010–8.0419 *μ*M. In other cases, major flavone formononetin** (4)** showed a moderate IC_50_ value of 13.4 *μ*M for evaluation against SH-SY5Y cell line* in vitro*, as compared to the higher IC_50_ value of 11.2 *μ*M for isoflavene odoriflavene** (52)**, or with the lower levels of 28.5 and 32.5 *μ*M for (3*R*)-5′-methoxyvestitol** (45)** and 2′-*O*-methyl-isoliquiritigenin** (66)**, respectively [25].

### 4.2. Antioxidant Activities

Frequently, naturally occurring phenols and flavonoids not only were shown to be major components in the genus* Dalbergia* but also have been the focus of biological assays [12]. The several therapeutic uses of medicinal plant* D. odorifera* species might be related to mostly flavonoids, especially in terms of the possible role of flavonoids in the prevention of oxidative stress. Admittedly, the potential antioxidant activity of flavonoids was figured out by chelating with metal ions, which therefore prevented their participation in free radical generation reactions [25, 59]. In an extensive research to identify the antioxidant bioassay of isolated flavonoids from heartwood of* D. odorifera* species, a flavanone eriodictyol** (22)** and a neoflavone 3′-hydroxymelanettin** (56)** exhibited stronger activity than commonly used synthetic antioxidant butylated hydroxytoluene (BHT), as far as compounds** 16-17**,** 23-24**, and** 35** in all three methods: oil stability index (OSI), potassium ferricyanide reducing power, and 2,2-azinobis(3-ethylbenzothiazoline-6-sulfonate) radical (ABTS^•+^) scavenging [33]. To take the concentration 0.012% and OSI method as an example, compounds** 22** and** 56** established the antioxidant protection factor (Pf) values of 6.48 and 4.20, respectively, whereas compounds** 16-17**,** 23-24**, and** 35** ranged from 1.09 to 1.13 (if Pf < 1, the sample had prooxidant activity; if Pf = 1, the sample had no antioxidant activity; if 1 < Pf < 2, the sample had antioxidant activity; if Pf > 3, the sample had strong antioxidant activity), as compared to this one of 3.61 of positive control BHT. As a result, the most striking feature in relation to structural characterization was the strong activity of compounds** 22** and** 56** due to* ortho*-hydroxyl unit in ring B, along with internal hydrogen bond between 5-hydroxyl group and carbonyl group C(4)=O which might eliminate catalytic oxidation performance of the trace amounts of metal ions [33]. Similarly, among nine tested components** 3**,** 8**,** 40**,** 44**,** 71**,** 99**, and** 129**–**131**, at the concentration of 0.02% or with 0.04% and 100°C, the Pf values of six of them** 3**,** 8**,** 40**,** 44**,** 71**, and** 99** were more than 3, while three unsaturated fatty acids** 129**–**131** had Pf values lower than 2 in the OSI method [19]. Particularly, a chemical component was found in heartwood and root; namely, (3*R*)-2′,3′,7-trihydroxy-4′-methoxyisoflavanone** (40)** displayed a Pf value 3-fold higher than positive control BHT and *α*-tocopherol in both concentrations 0.02% and 0.04%, which might be reasonable from three adjacent, two hydroxyl, and one methoxy groups [19, 20, 34].

In the third case of OSI model, at the concentration 0.1 mM, compounds** 4**,** 45**,** 52**,** 66**, BHT, and *α*-tocopherol had Pf of 2.79, 2.70, 3.31, 2.32, 4.21, and 3.72, respectively [25]. Meantime, at the concentration 0.2 mM and 100°C, the Pf values of 4.67, 3.30, 4.81, 3.50, 5.82, and 4.21 were nominated for the above compounds, respectively, or, with the concentration 0.1 mM adding Fe^3+^ (4 *μ*M) and at 100°C, all tested compounds** 4**,** 45**,** 52**, and** 66** were found to have Pf values of 0.9, 1.25, 1.96, and 1.79, respectively. Furthermore, compounds** 4**,** 45**,** 52**, and** 66** also showed inhibitory effects on the antioxidant systematic glutathione (GSH) level decrease of rat lens induced by UV irradiation comparable with positive control *α*-tocopherol [at the concentration 0.43 mM, a range of 27.1–29.7 for tested compounds and 26.2 *μ*g GSH/g tissue for positive control; at the concentration 0.86 mM, a range of 38.8–39.4 for tested compounds and 39.5 *μ*g GSH/g tissue for positive control] [25].

A poorly aqueous soluble chalcone butein** (68)** was precipitated out of methanol extract of* D. odorifera* species heartwood [18, 24, 46], which was chelated with metal ions Fe^2+^ (UV *λ*_max_: 286 and 422 nm) and Cu^2+^ (286 and 454 nm) and shown as a potential antioxidant agent with iron-induced lipid peroxidation inhibition in rat brain homogenate in a concentration-dependent manner with the IC_50_ value of 3.3 ± 0.4 *μ*M, IC_0.002_ value of 9.2 ± 1.8 *μ*M in DPPH reducing experiment (which was more significant than reference compound *α*-tocopherol 11.9 ± 0.2 *μ*M and BHT 14.5 ± 2.5 *μ*M), and IC_50_ value of 5.9 ± 0.3 *μ*M in xanthine oxidase-induced uric acid formation inhibitory activity [46]. Besides that, each molecule of compound** 68** scavenged the peroxyl radical derived from 1.4 molecules of 2,2-azobis(2-amidinopropane dihydrochloride) (AAPH) in aqueous phase, but not that from 2,2-azobis(2,4-dimethylvaleronitrile) (100 mM) in hexane, adding that this compound, which has been used as an inhibitor against Cu^2+^-induced thiobarbituric acid-reactive substance (TBARS) of human low-density lipoprotein (LDL) with an IC_50_ value of 6.3 ± 0.2 *μ*M and 30 *μ*M butein** (68)**, could reduce the electrophoretic change of oxidatively modified LDL [46]. However, at the concentration 100 *μ*M, this compound did not react with H_2_O_2_ (0.5–1.0 mM) and inhibited the hydroxyl radical-induced deoxyribose degradation [46].

### 4.3. Anti-Inflammatory Activities

Inflammation can be seen as a part of the complex biological response of body tissues to harmful stimuli, such as irradiation, physical damage, metabolic overload, or infection [60]. Nuclear factor-*κ*B (NF-*κ*B) activation has been playing a central role in inflammatory reactions [4, 37], while macrophages played an important role in regulating inflammatory responses via production of various proinflammatory cytokines and proinflammatory mediators, such as nitric oxide (NO), prostaglandins, tumor necrosis factor-*α* (TNF-*α*), and interleukin-1*β* (IL-1*β*) [4, 37, 43]. Additionally, nitric oxide synthase (iNOS) and cyclooxygenase-2 (COX-2) protein expressions also conducted production levels of NO and prostaglandin PGE2 [61]. Normally, lipopolysaccharide (LPS) was used as an inflammatory stimulant in the anti-inflammatory experiments as it induces NF-*κ*B activation through phosphorylation of I*κ*B inhibitor [4, 37, 61]. Hemeoxygenase-1 (HO-1) was an enzyme that catalyzed the degradation of heme to generate carbon monoxide, biliverdin, and free iron, and it also has been involved in the reduction of proneuroinflammatory mediators and inflammatory expressions [4, 5, 37, 43]. Therefore, HO-1 and its related by-products can be seen as the critical regulators of inflammation with macrophages acting as the critical targets [44]. Diseases related to inflammation include arthritis, hepatitis, septic shock syndrome, neuronal disorders caused by extensive and uncontrolled injuries, or irregular inflammatory responses [37, 61]. Among them, neurodegenerative diseases such as Alzheimer's, Parkinson's, or Huntington's disease have been increasing in recent decades, which was closely related to the activity of proinflammatory mediators, such as nitric oxide (NO) and prostaglandin E2 (PGE_2_) in microglia [61]. Consequently, anti-inflammatory drugs of medicinal plants have been receiving much more attention from researchers. For instance, more recently, the phytoconstituent plumericin from the Amazonian plant* Himatanthus sucuuba* improved as a new potential agent of NF-kB pathway in both anti-inflammatory* in vitro* and* in vivo *experiments [60].

We now present notes on several isolated compounds from* D. odorifera* species. First of all, a neoflavone derivative** 57** gave cell viability with the concentration range of 10–80 *μ*M, in which the amounts of NO, PGE2, TNF-*α*, and IL-1*β* production, iNOS and COX-2 expressions, IkB-*α* phosphorylation and degradation, NF-kB (p65) translocation, and NF-kB DNA-binding activity were reduced with increasing concentration of compound** 57** from 10 to 80 *μ*M in LPS (1 *μ*g/mL) stimulated primary murine peritoneal macrophages, whereas the same results were found in the concentration range of 5–40 *μ*M for 4,2′,5′-trihydroxy-4′-methoxychalcone** (67)** [37, 45]. In the comparison, compounds** 57** and** 67** offered HO-1 expression at the highest level with the conditions 80 *μ*M and 24 h and 40 *μ*M and 12 h treatment, respectively; meantime, HO-1 induction was observed to be evident at 6 h and reduced after 24 h when fixed at the concentrations for compounds** 57** (80 *μ*M) and** 67** (40 *μ*M) [37, 45]. With extensive research, tin protoporphyrin (SnPP) (50 *μ*M), a competitive inhibitor of HO-1 activity, partially reversed the inhibitory effects of latifolin** (57)** (40 or 80 *μ*M) on LPS-induced NO, PGE2, TNF-*α*, and IL-1*β* levels, along with compound** 57**, remarkably effected attenuation of I*κ*B-*α* degradation, NF-*κ*B translocation, and the DNA-binding activity of NF-*κ*B in the presence of SnPP, while the same happened to compound** 67** (40 *μ*M) [37, 45]. In addition, 80 *μ*M latifolin** (57)** gave increased nuclear Nrf2 levels and decreased cytoplasmic Nrf2 levels in 15–120 min treatment, as compared to 40 *μ*M 4,2′,5′-trihydroxy-4′-methoxychalcone** (67)** in 0.5–1.5 h treatment. Furthermore, compound** 57** gradually increased ARE luciferase activity in a dose-dependent manner from 10 to 80 *μ*M and stabilized at 120 min [37]. In addition, the role of Nrf2 in HO-1 expression was also studied using Nrf2 siRNA against Nrf2, in which transient transfection with Nrf2 siRNA completely suppressed HO-1 protein expression by compound** 67** (40 *μ*M) [45]. Second, isoliquiritigenin** (65)** did not show cytotoxicity for RAW 264.7 macrophages event at concentration 20 *μ*M, and at the concentration 10 *μ*M, compound** 65** displayed the results in inhibitory percentage of 86 and 79% for NO and IL-1*β* production when LPS (200 ng/mL) stimulated RAW 264.7 macrophages, respectively, and absolutely inhibited iNOS mRNA and protein and TNF-*α* mRNA expression [44]. In addition, the inhibition of LPS (200 ng/ml) induced NO and TNF-*α* production by compound** 65** (10 *μ*M) was related to its ability to induce HO-1 expression in RAW 264.7 macrophages in the presence or absence of 20 *μ*M SnPP [44]. Besides, we observed an increase in the levels of HO-1 mRNA and protein expression in RAW264.7 macrophages when examined with compound** 65** (1–10 *μ*M), while mitogen-activated protein kinases (MAPKs) signal inhibitory experiment confirmed that the induction of HO-1 by compound** 65** (10 or 20 *μ*M) was inhibited by ERK1/2 inhibitor U0126 but failed in selective JNK inhibitor SP600125 or p38 inhibitor SB203580 in a dose-dependent manner [44]. In the third case, a neoflavone 9-hydroxy-6,7-dimethoxydalbergiquinol** (60)** and two arylbenzofuran derivatives, (2*R*,3*R*)-obtusafuran** (119)** and isoparvifuran** (123)**, were derived from* D. odorifera* heartwood; the viability of cells incubated with various concentrations of neoflavone** 60** (5–50 *μ*M) and two arylbenzofurans** 119** and** 123** (1–20 *μ*M) was not affected significantly in BV2 microglia; compounds** 60 **and** 119** inhibited the levels of proinflammatory mediators NO, PGE_2_, TNF-*α*, and IL-1*β*, with the results of decreased iNOS and COX-2 appearing when BV2 microglia were stimulated by LPS at the doses of 500 ng/mL and 1 *μ*g/mL, respectively; however, compound** 123** did not show any decreased levels of the above proinflammatory agents and iNOS and COX-2 expressions event at concentration of 20 *μ*M [4, 5]. Hence, we paid further attention to compounds** 60 **and** 119**, in which two compounds** 60** (5–40 *μ*M) and** 119** (1–20 *μ*M) also revealed reduction in the levels of IkB-*α* phosphorylation and degradation, NF-kB (p65 and p50) translocation, and NF-kB DNA-binding activity in LPS at the doses of 500 ng/mL and 1 *μ*g/mL, respectively, stimulating BV2 microglia [4, 5]. As the same way of 4,2′,5′-trihydroxy-4′-methoxychalcone** (67)** in primary murine peritoneal macrophages, 9-hydroxy-6,7-dimethoxydalbergiquinol** (60)** and (2*R*,3*R*)-obtusafuran** (119)** possessed the highest HO-1 expression at the concentrations of 40 and 20 *μ*M, respectively, and also indicated increased nuclear Nrf2 levels and decreased cytoplasmic Nrf2 levels in 0.5–1.5 h treatment, and transient transfection with Nrf2 siRNA absolutely inhibited HO-1 protein expression in BV2 microglia [4, 5]. Followed by using an inhibitor of HO-1, SnPP (50 *μ*M) partially reversed the inhibitory effects of compound** 60** (40 *μ*M) on LPS (1 *μ*g/mL) induced NO, PGE2, TNF-*α*, and IL-1*β* levels and resembled the procedures of compound** 119** (20 *μ*M) when LPS (500 ng/mL) stimulated BV2 microglia [4, 5]. By far, utilizing 3-(4,5-dimethylthiazol-2-yl)-2,5-diphenyl tetrazolium bromide salt (MTT) in the assay of cell viability, activated microglia-mediated cell death of mouse hippocampal HT22 cells was significantly repressed by compound** 119** (1–20 *μ*M) after 24 h incubation [4]. In a continued case, at the noncytotoxic concentrations (10–80 *μ*M), phytoconstituent** 31** reduced the amounts of NO, PGE2, TNF-*α*, IL-1*β*, iNOS, and COX-2 protein inmunocontents using LPS (0.5 *μ*g/mL) stimulated BV2 microglia, which were identical to the procedures of compounds** 60** and** 119** [43]. At the highest nontoxic concentration 80 *μ*M of compound** 31**, HO-1 expression reached the highest level in either HT22 cells or BV2 microglia, and the time course of HO-1 induction evidently revealed that protein was first detectable 6 h after treatment, peaked around 18 h, and reduced after 12 h in both cells [43]. Possibly, this was similar to cases of compounds** 60 **and** 119**; SnPP (50 *μ*M) has been playing as an inhibitor for reversing the inhibitory effects of compound** 31** (80 *μ*M) on HO-1 induction in two cells, as well as proinflammatory mediators NO, PGE_2_, TNF-*α*, and IL-1*β* production in the experiment of using LPS (0.5 *μ*g/mL) stimulated BV2 microglia [4, 5, 43]. Twenty-six flavonoids** 4**–**6**,** 18-19**,** 24**,** 26**–**28**,** 35**–**37**,** 39**,** 41-42**,** 55**–**57**,** 61-62**,** 65**, and** 68**–**70** and one xanthone derivative** 128 **were isolated from ethyl acetate soluble fraction of* D. odorifera* heartwood by following their potential to inhibit the LPS-induced nitric oxide production in RAW 264.7 cells [24]. Among them, (2*S*)-pinocembrin** (24)** showed the most potent inhibitory activity with the IC_50_ value of 18.1 *μ*M due to the lack of hydroxyl groups at the B-ring but it had a 5-hydroxyl group at the A-ring. Meanwhile, compounds** 4**,** 18-19**,** 26**,** 55**–**57**,** 58**,** 61-62**,** 65**,** 68**, and** 71** had IC_50_ values of 56.1, 45.5, 43.7, 53.5, 53.2, 45.5, 73.2, 74.0, 73.9, 70.3, 72.0, 35.1, and 83.7 *μ*M, respectively, while the IC_50_ values of the remaining ones were obtained more than 100 *μ*M, as compared to a positive control compound aminoguanidine (IC_50_ value of 16.6 *μ*M) [24].

Leukotrienes (LTs) C_4_, D_4_, and E_4_, are members of lipid mediators formed by the 5-lipoxygenase pathway of arachidonic acid metabolism; in addition, leukotrienes were involved in bronchoconstriction, inflammation, microvascular permeability, and mucus secretion in asthma and chronic obstructive pulmonary diseases [62]. Inflammatory aspects, neutrophils, mast cells, and macrophages usually possessed production of leukotrienes to promote inflammatory diseases [39]. In current surveys of potential leukotriene inhibitors from medicinal plant* D. odorifera *species, methylene chloride and chloroform extracts exhibited inhibitory ability of LTC_4_ production in AB-CXBG Mct-1 mastocytoma cells with IC_50_ values of 0.52 (80%) and 3.0 *μ*g/ml, respectively, while two compounds** 71** and** 124 **were obtained from chloroform extracts that displayed LTC_4_ inhibitory activity with the IC_50s_ values of 0.5 and 0.05 *μ*M [39]. Besides, arylbenzofuran derivative** 124** acted as a specific inhibitor of 5-lipoxygenase with an IC_50_ value of 0.08 *μ*M against the soluble rat enzyme; however, it was inactive against cyclooxygenase [39].

Regarding cytoprotection of HT22 cells by antioxidative agents, glutamate cytotoxicity was responsible for the accumulation of reactive oxygen species and was closely related to neuronal degeneration in central nervous system diseases, for instance, epilepsy and ischemia [28, 43]. Glutamate toxicity induced neuronal cell death via both receptor-initiated excitotoxicity and non-receptor-mediated oxidative stress [28, 43]. As far as we know, the immortalized mouse hippocampal HT22 cells have shown good advances for studying oxidative glutamate toxicity due to the fact that they were similar to neuronal precursor cells but lacked functional ionotropic glutamate receptors, hence excluding excitotoxicity as a cause for glutamate triggered cell death [43]. The glutamate-induced oxidative injury in HT22 cells model was also applied in these researches of isolated compounds from* D. odorifera *heartwood, including seventeen compounds** 10**,** 30**–**33**,** 57**–**60**,** 65**,** 67**,** 97-98**,** 119**,** 123**, and** 126-127** [28]. Herein, two new compounds, a flavan derivative (2*S*)-6,7,4′-trihydroxyflavan** (32)** and a chalcone derivative 4,2′,5′-trihydroxy-4′-methoxychalcone** (67)**, along with nine known compounds** 10**,** 31**,** 33**,** 57**–**60**,** 123**, and** 126**, displayed protective effects with EC_50_ values in the range of 2.85–25.79 *μ*M, especially in terms of compounds** 31** (EC_50_ value of 3.3 *μ*M),** 33** (2.85 *μ*M),** 57** (5.82 *μ*M),** 59-60** (6.54 and 8.14 *μ*M, resp.),** 67** (7.47 *μ*M),** 123** (3.09 *μ*M), and** 126** (8.54 *μ*M) that were more potent than the positive control trolox (15.8 *μ*M), while the remaining ones established an insignificant EC_50_ value of more than 50 *μ*M [28]. Noting compound** 31**, at the noncytotoxic concentration range of 10–80 *μ*M, 6,4′-dihydroxy-7-methoxyflavanone** (31)** showed potent protective effects on glutamate-induced cytotoxicity and reactive oxygen reaction production with EC_50_ values of 26.3 and 22.4 *μ*M, respectively [43].

Activated neutrophils release lysosomal enzymes and generate highly reactive oxygen species [18]. The number of lysozymes secreted by stimulated rat neutrophils tended to exceed the amount of *β*-glucuronidase [63]. The uncontrolled release of *β*-glucuronidase and lysozymes may deleteriously injure adjacent cells [18]. With analysis of the results of Sprague-Dawley rat neutrophil degranulation and superoxide formation experiments, cearoin** (97)** acted as an inhibitor for both *β*-glucuronidase and lysozyme release with significant IC_50_ values of 7.9 and 11.7 *μ*M, respectively, as compared to a reference compound trifluoperazine (significant IC_50_ values of 16.9 and 12.8 *μ*M, resp.), compound** 68** (significant IC_50_ value of 16.8 *μ*M against the lysozyme release), and compound** 125** (significant IC_50_ value of 20.6 *μ*M for inhibition of the release of *β*-glucuronidase), whereas koparin** (12)**, bowdichione** (20)**, and (*S*)-4-methoxydalbergione** (125)** inhibited superoxide formation induced by phorbol myristate acetate (PMA) from rat neutrophils with significant IC_50_ values of 1.9, 0.9, and 4.9 *μ*M, respectively, and xenognosin B** (13)** and 3′-*O*-methylviolanone** (37)** showed IC_50_ values of 6.2 and 3.0 *μ*M, respectively, to suppress superoxide formation induced by formyl-Met-Leu-Phe-OH (FMLP) [18]. In the mast cell degranulation experiment, the order of IC_50_ values of 17.6, 17.9, 22.3, 53.7, and 71.6 *μ*M was assignable to compounds** 125**,** 97**, positive control mepacrine,** 77**, and** 20**, respectively, against the release of *β*-glucuronidase [18]. Additionally, these compounds also provided evidence on antiallergic activity, with IC_50_ values of 16.3, 20.0, 37.1, 51.1, and 14.7 *μ*M for compounds** 97**,** 125**,** 77**,** 20**, and positive control mepacrine, respectively [18]. It was therefore assumed that cearoin** 97** and (*S*)-4-methoxydalbergione** (125)** could be antiallergic agents [18].

### 4.4. Antibacterial Activities

On the basis of screening results against the motility and viability of phytopathogenic* Aphanomyces cochlioides *zoospores, not only were three flavonoid derivatives** 4**,** 50**, and** 71** separated from acetone extract of medicinal Chinese plant* D. odorifera* heartwood, but also medicarpin** (71)** showed repellent activity at 150 *μ*g/ml, while claussequinone** (50)** and formononetin** (4)** showed stimulating and attracting activity at 100 and 50*μ*g/ml, respectively; in the meantime, significantly, the constituent that contained a mixture of three (1 : 1 : 1, w/w/w) had advantages in repellent activity at 50 *μ*g/ml [26].

The isolated compounds** 21**,** 29**,** 35**,** 38**,** 40**-**41**,** 44**,** 65**, and** 122** were further tested* in vitro *for antibacterial activity against* Ralstonia solanacearum *strain by the filter paper disc agar diffusion method [34]. The results were presented as diameters of inhibition zones in mm. Among the records, (3*R*)-vestitone** (38)** established the strongest activity with 16.62 mm, which could be approximated by positive control streptomycin sulfate (16.80 mm); meantime, the lowest value of 6.53 mm was assignable to (3*R*)-sativanone** (35)**. According to this paper, the reasonable mechanism of decreased activity was due to the absence of the 2′-OH group in compound** 35**, whereas the increased activity in compound** 38** was caused by the lack of carbonyl group C(4)=O in the C-ring [34]. In the same manner, three compounds** 73**,** 92**, and** 120** showed, against* R. solanacearum*, inhibition zone diameters of 10.03, 10.55, and 14.15 mm, respectively, at the concentration of 5.0 mg/mL when compared to that of positive control kanamycin sulfate with inhibition zone diameter of 28.38 mm [7]. Meanwhile, those for other tested compounds** 43**,** 72**, and** 93** were 8.02, 8.47, and 7.13 mm, respectively, at the concentration of 10 mg/mL, as well as the inactive results for** 73-74**,** 76-77**, and** 79** [7].

We now present additional information about the antibacterial assay. Wang et al. (2014) identified the presence of fifteen sesquiterpenes** 102**–**112** and** 115**–**118** in the heartwood, in which bioassay results displayed that both compounds** 102-103 **had inhibitory effects on* Candida albicans* with 9.21 and 10.86 mm, respectively, together with compound** 102** exhibiting inhibitory activity against* Staphylococcus aureus* with 11.02 mm; the thirteen remaining ones did not show activity when compared to those of positive controls fluconazole (*C. albicans*, 30.64 mm) and kanamycin sulfate (*S. aureus*, 24.52 mm) [41].

### 4.5. Antithrombotic and Platelet Activities and Prostaglandin Biosynthetic Enzyme Inhibition (PG Synthetase Inhibition)

The antithrombotic procedure, which is concerned with antiplatelet and anticoagulant therapies, prevented and treated blood coagulation processes, cardiovascular disorders, rheumatoid arthritis, hyperuricemia, chronic stable angina, stroke, and various inflammatory conditions [42, 64]. With the screening of potential antithrombotic agents, for instance, aspirin and clopidogrel have been playing well-known roles in preventing adverse cardiovascular events in patients [65]. Herein, two new sesquiterpenes** 113-114** indicated three golden criterions—activated partial thromboplastin time (APTT), thrombin time (TT), and prothrombin time (PT)—for evaluating the blood coagulation process like control group [saline with identical amount of Tween 20 (0.05 ml/10 mL)] at the concentrations of 1, 10, and 100 *μ*g/mL [42]. Meantime, the inhibition percentage of antiplatelet assay scored about 50% for both compounds** 113-114** at the concentration of 10 *μ*mol/mL, whereas at the middle concentration 5 *μ*mol/mL, compounds** 113-114** accounted for 40 and 25%, respectively [42].

With extensive researches, Goda et al. (1985 and 1992) suggested that platelet aggregation and PG synthetase inhibitors greatly were involved in the balance between vasoconstrictor thromboxane A2 (TXA2) and vasodilator prostacyclin (PGI_2_) [21, 22]. Herein, compounds** 44**,** 48-49**,** 52**,** 94**–**96**, and** 100** reached IC_50_ values of 47, 110, 63, 4.8, 7.7, 9.2, 2.8, and 23 *μ*M for inhibiting PG synthetase, respectively [21, 22]. Obviously, compounds** 52**,** 94**–**96**, and** 100** possessed a significant IC_50_ value with a comparable status to that of potent inhibitor indomethacin with an IC_50_ value of 4.9 *μ*M [21, 22]. Regarding the inhibition of rabbit platelet aggregation, only three compounds** 95-96** and** 100 **strongly inhibited platelet aggregation induced by arachidonic acid (128 *μ*M) and collagen (20 *μ*M), whereas they showed insignificance with inducer adenosine diphosphate (10 *μ*M) [21, 22].

### 4.6. Antiosteosarcoma and Antiosteoporosis

As a part of ongoing effort to look for natural products with anticancer effects* in vitro* and* in vivo*, recently, Park et al. (2016) mentioned the role of 4-methoxydalbergione** (125)** in suppressing growth and inducing apoptosis in human osteosarcoma cells in* in vitro *and* in vivo *xenograft models through downregulation of the JAK2/STAT3 pathway [48]. The presence of compound** 125** improved the significant inhibitory effects on cell growth of both osteosarcoma MG63 and U-2-OS cells with concentration-dependent manners at 1, 10, and 30 *μ*M for 24, 48, and 72 h; in fact, by the treatment of compound** 125**, morphologic images importantly showed that the MG63 cells were gradually reduced in size and changed into a small round single cell shape compared to U-2-OS cells [48]. 4-Methoxydalbergione** (125)** also suppressed the proliferation of osteosarcoma cells and induced apoptosis as evidenced by Annexin V^+^ and TUNEL^+^ cells; meanwhile, this apoptosis, on the one hand, possessed upregulation of apoptotic proteins procaspase-3 and PARP and, on the other hand, wasaccompanied with downregulation of antiapoptotic proteins Bcl-2, Bcl-xL, and survivin in MG63 cells [48]. In addition, quinone derivative** 125** directly inhibited the phosphorylation of JAK2 and the downstream phosphorylation of STAT3, especially maximum inhibition occurring at the concentration of 30 *μ*M, together with this compound inducing the reducible activation of ERK1/2, JNK, p38 MAPK, and cAMP response element binding protein (CREB) in a dose-dependent manner, but it caused a concentration-dependent increase of regular JAK2/STAT3 signaling factor phosphatase and tensin homolog deleted on chromosome ten (PTEN) in osteosarcoma cells [48]. Last but not least, compound** 125** reduced colony formation in soft agar and inhibited tumor growth, such as 9 mg/kg; this one significantly decreased by 22.25 ± 11.46% of the tumor weight compared to control in BALB/c athymic nude mice xenograft model in association with the reduced expression of proliferating cell nuclear antigen (PCNA), proliferation marker (Ki67), therapeutic target molecule (p-STAT3), and antiapoptotic molecule (survivin) in tumor tissues [48].

As mentioned above, a novel chalcone** 67** named 4,2′,5′-trihydroxy-4′-methoxychalcone was isolated from* D. odorifera* heartwood, and its biological assays involved either the protective effects against glutamate-induced oxidative in HT22 cells or anti-inflammatory properties by inducing HO-1 in murine macrophages [28, 45]. In further surveys, with noncytotoxic concentrations increasing from 0.1 to 1.0 *μ*M, biological active chalcone** 67** dose-dependently stimulated osteoblastic differentiation, as generated by growing the indices of alkaline phosphatase activity (ALP) and ALP staining for 5 days, Alizarin Red S staining for 7 and 14 days (the degree of mineralization), and the levels of mRNAs encoding the bone differentiation markers, including ALP, bone sialoprotein (BSP), osteopontin (OPN), and osteocalcin (OCN) for 7 days [38]. Likewise, compound** 67** (0.1–1.0 *μ*M) activated bone morphogenetic protein (BMP) signaling pathway through upregulating the expression of* Bmp*2 and* Bmp*4 genes and the protein level of phospho-Smad1/5/8 for 2 days, as well as Wnt/*β*-catenin signaling pathway; this compound treatment showed the increased expressions of Wnt1 and Wnt3 mRNA (but not Wnt5), phosphorylation of GSK3, and the expression of *β*-catenin proteins N and T for 2 days, in addition to the fact that the treatment of osteoblasts with this compound did not affect MAPKs signaling pathway (the phosphorylation of ERK1/2, JNK, and p38 MAPK) for 30 min [38]. The BMP antagonist, 10 *μ*g/mL noggin, pretreatment drastically inhibited compound** 67**-mediated ALP activity and ALP staining for 5 days and mineralized nodule formation (Alizarin Red S staining) for 14 days, whereas the Wnt inhibitor, 0.5 *μ*g/mL Dickkopf-1 (DKK1), was partially attenuated [38]. Followed by the author's view, runt related transcription factor 2 (Runx2) integrated the BMP2 and Wnt/*β*-catenin signaling pathways in the regulation of osteoblastic differentiation; therefore, the results of this extensive research displayed that Runx2 expression was significantly increased by a chalcone derivative** 67** (0.1–1.0 *μ*M) after 48 h incubation, in addition to having DKK1 (0.5 *μ*g/mL) and noggin (10 *μ*g/mL) attenuated** 67** stimulated Runx2 protein expression for 2 days, while Runx2 siRNA (directly downregulates Runx2), Smad4 siRNA (a final molecule of BMP2-Smad1/5/8 pathway), and TCF1 siRNA (a final molecule of Wnt/beta-catenin pathway) decreased** 67**-induced ALP activity for 5 days [38].

### 4.7. Vasorelaxant Activities

As far as we know, secondary messengers such as cyclic adenosine monophosphate (cAMP) and cyclic guanosine monophosphate (cGMP) are currently the most well documented cyclic nucleotides which were used for intracellular signal transduction in many different organisms. In most cases, the elevation of cAMP and cGMP concentrations possessed independent inhibition of vascular smooth muscle cell proliferation [47, 66]. The intracellular concentrations of cAMP and cGMP were identified by their rate of formation through agonist induced stimulation of adenylate and guanylate cyclases [47]. Phosphodiesterases (PDEs) are a family of enzymes that break phosphodiesterase bonds and hence play a central role in regulating intracellular levels of the second messengers cAMP and cGMP [67]. PDEs I, II, and III can utilize both cAMP and cGMP as a substrate, while numerous cyclic nucleotide phosphodiesterase isoenzymes PDEs I, III, IV, and V act as degradable agents of cGMP by hydrolysis [47]. It is therefore suggested that selective phosphodiesterase inhibitors have been playing roles in relaxing smooth muscles. In the current review paper, butein** (68)** also participated in investigating the phenylephrine precontracted rat aorta by measuring tension, cAMP and cGMP levels, adenylate and guanylate cyclases, and phosphodiesterase isoenzyme activities [47]. First, in the results, latent relaxation of compound** 68** on rat aortic rings precontracted with various vasoconstrictors, phenylephrine (3 *μ*M), KCl (60 mM), U-46619 (1 *μ*M), endothelin-1 (1 nM), and angiotensin II (3 *μ*M), received EC_50_ values of 7.4 ± 1.6, 10.5 ± 2.3, 14.3 ± 3.3, 11.8 ± 2.0, and 13.6 ± 3.7 *μ*M, respectively. Second, this isolated compound with the concentration range of 1–100 *μ*M caused endothelium-dependent relaxation of rat aorta precontracted with phenylephrine, but it was abolished in endothelium denuded aorta and in endothelium in the presence of 300 *μ*M N^G^-monomethyl-l-arginine (l-NMMA, an inhibitor of l-arginine nitric oxide), 10 *μ*M oxyhemoglobin (bound to nitric oxide), and 50 *μ*M methylene blue (a soluble guanylate cyclase inhibitor); however, this effect was unchanged by 10 *μ*M indomethacin or 100 nM charybdotoxin. It was clearly shown that the vasorelaxant effect of compound** 68** is dependent on endothelium and was mediated by endothelium derived relaxing factor. Third, compound** 68** (1–100 *μ*M) increased both cAMP and cGMP in the state of incubation between this compound and endothelium intact aorta; for example, butein (100 *μ*M) caused a 4.1 ± 0.3- and 2.9 ± 0.3-fold increase in rat aorta, respectively. Fourth, using diethylaminoethyl- (DEAE-) Sephacel chromatography, four phosphodiesterase forms were isolated from rat aorta, in which cAMP-specific PDE-IV was potently inhibited by butein** (68)** and rolipram with IC_50_ values of 10.4 ± 0.4 and 3.2 ± 0.2 *μ*M, respectively; typical PDEs I, III, and IV were controlled by compound** 68 **with an IC_50_ value of more than 100 *μ*M. Fifth, adenylate and guanylate cyclases levels did not change by 30 or with 100 *μ*M butein** (68)**. Last but not least, in the absence of endothelium, coadministration of 0.01 *μ*M cAMP-isoprenaline (a *β*-adrenoceptor agonist) or with 0.01 *μ*M cAMP-forskolin (an adenylate cyclase activator) did not significantly modify the effects elicited by butein** (68)**, but 1 nM cGMP-sodium nitroprusside (a soluble guanylate cyclase activator) or 0.01 *μ*M cGMP-trequinsin (a phosphodiesterase III inhibitor) still enhanced the relaxant effects of butein** (68)**, and 0.1 *μ*M of isoprenaline and forskolin still weakly but significantly enhanced the relaxant effect of butein** (68)** in endothelium denuded aorta; however, in the presence of endothelium, all tested components gave potent effects on the relaxant activities of butein** (68)**.

### 4.8. Alpha-Glucosidase Inhibitory Activities

As can be seen, alpha-glucosidase inhibitors were used in the treatment of patients with diabetes mellitus type 2 due to reduction of the impact of carbohydrates on blood sugar. Pseudotetrasaccharide acarbose, with brand name Precose, has been employed as an alpha-glucosidase inhibitor for managing diabetes mellitus type 2. Naturally occurring flavonoids** 10**,** 21**,** 24**,** 33**,** 35**,** 37**,** 57**, and** 65** were assessed to evaluate *α*-glucosidase inhibitory activity when acarbose was used as a positive control [29]. The result showed that an isoflavone 7,3′-dihydroxy-5′-methoxyisoflavone** (10)**, two flavanones (2*S*)-liquiritigenin** (21)** and (2*S*)-6,4′-dihydroxy-7-methoxyflavan** (33)**, and a chalcone isoliquiritigenin** (65)** possessed significant IC_50_ values of 8.43, 3.75, 4.43, and 0.96 mg/mL to compare with acarbose (IC_50_ value of 5.08 mg/mL). Similarly, compounds** 4**,** 11**,** 21**,** 45**,** 49-50**, and** 71 **exerted potential inhibition upon yeast alpha-glucosidase in the following order: formononetin** (4)** (IC_50_ value of 0.51 mM) > medicarpin** (71)** (2.93 mM) > tectorigenin** (11)** (3.52 mM) > liquiritigenin** (21)** (3.61 mM) > acarbose (9.11 nM) > mucronulatol** (48)** (12.53 mM) > (3*R*)-calussequinone** (50)** (29.38 mM) > (3*R*)-5′-methoxyvestitol** (45)** (>30 mM) [3].

In view of all the circumstances, parallel with the phytochemical isolation, the biological studies on isolated compounds are diverse. Herein, the biological experiments of isolated constituents were compiled in Table 2.

### 4.9. Biological Activities of Extracts

Pharmacological screening of methanol extract has generally been shown to be the first choice in almost all natural product researches. As expected, here, 60% methanol extract of Chinese medicinal plant* D. odorifera *heartwood showed potent repellent activity against* A. cochlioides *zoospores at the dose of 200 *μ*g/mL [26].

This was followed by the assessment of* D. odorifera *heartwood extracts for experimental against human tumor cell lines MES-SA, MES-SA/DX5, HCT-15, and HCT15/CL02, in which the significant EC_50_ values for all tested cell lines were in the range of 7.8–11.3 *μ*g/mL for methanol extract and 5.5–10.0 *μ*g/mL for ethyl acetate fraction; meantime, the ranges of 34.2–60.4 *μ*g/mL and more than 100 *μ*g/mL were assignable to* n*-butanol and water fractions, respectively [23].

Regarding the inhibitory effects of* D. odorifera* heartwood extracts on the release of *β*-glucuronidase and lysozyme from rat neutrophils, the highest percentages of *β*-glucuronidase and lysozyme inhibition were 75.5 and 87.2%, which accounted for* n*-butanol extract at the dose 100 *μ*g/mL, and the lowest rates of 29.9 and 19% were for the water layer at the dose of 30 *μ*g/mL; plus, at the experiment doses of 30 and 100 *μ*g/mL, the methanol extract displayed significant percentages of inhibition (more than 67%) for both cases [18]. At the dose of 100 *μ*g/mL, either methanol or ethyl acetate extracts strongly took part in suppressing the amount of *β*-glucuronidase release (6.9 and 16.8 *μ*g/mL, resp.) in antihistamine from mast cells, whereas it was in sharp contrast with ethyl acetate and water extract (9.7 and 14.2 *μ*g/mL, resp.) at the dose of 10 *μ*g/mL [18].

Up to now, there is only one report by Lianhe et al. (2012) studying the* in vitro *antioxidant activity of* D. odorifera* seed oils; the methanol extract of these oils can be held responsible for measurements of DPPH (1,1-diphenyl-2-picrylhydrazyl) radical scavenging, ferric ion reducing power, ferrous ion chelating activity, and linoleic acid peroxidation inhibition models [58]. Indeed, it was described that the DPPH radical-scavenging activity of 12.5 mg oils extract was equal to 0.170 mg gallic acid (1 mM), with reducing power and ferrous ion chelating abilities of oils in the tested range of 3.12–50 mg oil equivalent/mL increasing with increasing the concentration, but reducing power was relatively weaker than a reference compound gallic acid (1 mM), while the chelating capacity of oils extract and a reference compound ethylenediaminetetraacetic acid (EDTA) was calculated as 54.97 and 46.54% at 50 mg oil equivalent/mL and 0.1 mM, respectively, together with the oils extract which was a potential antioxidant in preventing lipid peroxidation such as 3.12 mg oil equivalent/mL that gave higher inhibition of lipid peroxidation than gallic acid (1 mM) during storage [58].

As a result of DPPH radical-scavenging activity, in general, the EE, PE, BE, and WE extracts increased the ability against DPPH agent with increasing concentration from 0.1 to 0.8 mg/mL, and with regard to the low concentration, such as the concentration of 0.1 mg extract/mL, the radical-scavenging activities of WE, PE and BE were much less than that of EE extract (28.7 ± 0.2, 22.7 ± 0.9, 19.3 ± 2.7, and 43.1 ± 0.7%, resp.), but they were close at the high concentration of 0.8 mg extract/mL (59.5 ± 1.0, 67.1 ± 0.2, 57.2 ± 1.1, and 62.8 ± 0.5%, resp.); however, these were not significant when compared with positive control vitamin C (Vit. C) at the concentration of 0.1 mg/mL [57]. Reducing power, linoleic acid peroxidation inhibition, and chelating ability on ferrous ions of* D. odorifera *seed extracts are dependent on the concentrations; generally, these items will increase with increasing concentrations from 0.1 to 1.0 mg/mL for reducing power and linoleic acid peroxidation inhibition and from 0.5 to 2.0 mg/mL for chelating ability on ferrous ions [57]. In addition, it was noticeable to find that reducing power and linoleic acid peroxidation inhibition of different seed extracts revealed the following order: EE > WE > BE > PE, while this one for chelating ability on ferrous ions was WE > PE > BE > EE. Take the concentration at 1.0 mg/mL as an example; the reducing powers of the PE, EE, BE, and WE extracts and Vit. C were 0.351 ± 0.017, 1.230 ± 0.034, 0.444 ± 0.014, and 0.818 ± 0.006 and 2.408 ± 0.012, respectively, and the percentage of linoleic acid peroxidation inhibition of EE was 64.4 ± 2.1% which was higher than those of PE (14.0 ± 1.0%), BE (30.1 ± 1.4%), and WE (48.5 ± 1.7%) but lower than that of BHT (97.9 ± 2.5%), while, for instance, at 0.5 mg/mL, chelating abilities of the PE, EE, BE, and WE extracts and Na_2_EDTA were 18.6 ± 2.2, 6.8 ± 0.4, 9.9 ± 3.9, 64.4 ± 0.5, and 96.4 ± 1.5%, respectively [57]. This was similar to patterns of DPPH free radical scavenging at low concentration, reducing power, and linoleic acid peroxidation inhibition; the EE extract also exhibited higher antioxidant activity (which was equal to the ability of BHT during the storage) than the remaining ones in the Schaal oven test method; according to the author, regarding the EE extract, phenolic and flavonoid contents had a great influence on the DPPH free radical scavenging, reducing power, and linoleic acid peroxidation inhibition and no correlation of chelating ability, especially in terms of enriched compound with *t*_*R*_ 21.76 min and* m/z* 373.2 [M−H]^−^ in a total of 23 detectable flavonoids by using LC-MS method [57].

Zhang et al. (2011) reported DPPH radical-scavenging assay for* D. odorifera* leaves extracts, in which NPCE, HRE, and USE extracts and ascorbic acid revealed IC_50_ values of 0.194, 0.211, 0.239, and 0.122 *μ*g/mL, respectively; meantime, with regard to the ferric reducing ability of plasma (FRAP) method, the FRAP value for NPCE was 2.027 mmol FeSO4/g DW which was higher than those for HRE (1.893 mmol FeSO4/g DW) and USE (1.679 mmol FeSO4/g DW) [56]. Likewise, according to the DPPH radical-scavenging assay in the work of Ma et al. (2013), comparable results in the differential technical methods were found, where MA-ATPE, MAE, and HRE extracts generated notable concentration-dependent antioxidant activity with IC_50_ values of 0.342, 0.389, and 0.411 mg/mL, respectively [14].

Taking PG synthetase inhibition into consideration, the hot aqueous extract of* D. odorifera* heartwood was found to resist PG biosynthesis by 98% at the concentration of 750 *μ*g/mL, while the inhibitory effects of methanol extract, hexane, chloroform, and water fractions on this were 70, 97, 99, and 99% at the concentration of 150 *μ*g/mL respectively; in addition, benzene soluble fraction from chloroform fraction was associated with PG biosynthesis inhibition by 96% and 60% at the concentrations of 150 and 20 *μ*g/mL, respectively [21, 22].

Recently, Choi et al. (2017) reported the effects of a 60% ethanol extract of* D. odorifera* heartwood (DOE) on proinflammatory cytokine high mobility group box 1 (HMGB1) release in LPS treated murine RAW264.7 cells and a mouse model of endotoxemia [53]. With regard to the noncytotoxic concentration range of 0.1–10 *μ*g/mL, DOE extract dose-dependently suppressed the release of HMGB1 for 24 h and even up to 9 h after LPS (100 ng/mL) treatment and decreased cytosolic translocation of HMGB1 in murine RAW264.7 cells for 24 h [53]. DOE extract modulated HMGB1 via NO signaling based on NO level and iNOS expression that were markedly reduced in LPS stimulated RAW 264.7 cells for 24 h; even the significant inhibitory effect of DOE (0.1–10 *μ*g/mL) on the NO level was sustained up to 12 h after LPS (100 ng/mL) treatment [53]. With research on involvement of MAPK signaling cascade in LPS triggered release of HMGB1, in accordance with these findings, DOE (10 *μ*g/mL) extract significantly inhibited LPS (100 ng/mL) induced phosphorylation of JNK, but not the ERK and p38 pathways. According to this article, the injection of endotoxin generated an increase in the mortality rate of mice, which also correlated with the release of HMGB1 [53]. Notably, administration of DOE extract (20 mg/kg) conferred protection of BALB/c mice against lethal endotoxemia even up to 6 h after LPS (10 mg/kg) treatment, and late deaths of DOE-treated animals were not detected during the 2 weeks after LPS injection, whereas the same dose of DOE extract decreased the level of circulating HMGB1 that was observed by immunoblot analysis of sera collected at 20 h after LPS injection (10 mg/kg) [53].

Studying herbal cosmetics for preventing skin photoaging, 40, 60, 80, or 100% ethanol extracts of Korean medicinal plant* D. odorifera* heartwood and their constituents sativanone** (35)** and dalbergin** (53)** exerted an increased expression of type I collagen in human dermal fibroblasts with increasing doses from 0.1 to 10 *μ*g/mL, especially in terms of 60% ethanol extract [52]. As a consequence, 60% ethanol extract was further assessed to examine the effects on the expression of other extracellular matrix (ECM) related proteins in human dermal fibroblasts, in which this and its constituents** 35** (1 *μ*g/mL) and** 53** (10 *μ*g/mL) elevated the levels of type I collagen, elastin, and transforming growth factor-*β*1 (TGF-*β*1) for 38 h treatment. Similarly, the possible anti-skin-aging factor, 60% ethanol extract, also ameliorated the expression of type I collagen, elastin, and TGF-*β*1 in mouse skin exposed to UVB light (280–320 nm). To continue, 60% ethanol extract prevented effects of photoaging and maintained skin integrity by either reduction of metalloproteinase-2 (MMP-2) or upregulation of tissue inhibitors of metalloproteinase (TIMP-2 and TIMP-3) in skin tissue exposed to UVB light. Finally, in the experiments of 6-week-old hairless mice (HR-1) exposed to UVB light, 60% ethanol extract attenuated wrinkle formation and increased epidermal/dermal thickness. Possibly, it is concluded that* D. odorifera *extracts and their components may serve as therapeutic agents for photoaging when skin is damaged by chronic solar irradiation.

Angiogenesis was the physiological process via which new blood vessels formed from preexisting vasculature [68–70]. Angiogenesis is a normal and crucial process in growth and development, particularly in wound healing and in the formation of granulation tissue; for instance, promoting angiogenesis effects in the ischemic aspect can be considered a therapeutic option for treating diffuse coronary artery disease [70]. Meantime, in contrast, the birth of new blood capillaries in tumor cells has also accelerated the growth rate of solid tumors [70]. The Chinese medicine “Jiangxiang,” derived from* D. odorifera* heartwood, was used to treat various ischemic diseases such as trauma and injuries [7, 70]. Consequently,* D. odorifera* and other related species prompted us to conduct in-depth investigation on the angiogenic property [69, 70]. Herein, current clinical angiogenesis therapy research introduced the notion that* D. odorifera *ethanol extract (DOE) promoted angiogenic effects in both* in vivo *transgenic zebrafish and* in vitro* human umbilical vein endothelial cell (HUVEC) [70]. In the* in vivo *study, at first, the higher concentrations of 3–10 *μ*g/mL DOE significantly and dose-dependently promoted the zebrafish subintestinal vessels (SIVs) area to sprout new capillaries. Tg(fli1:EGFP)y1 transgenic embryos were incubated with 1, 3, and 10 *μ*g/mL of DOE from 24 to 72 hours after fertilization (hpf)). Second, 3–10 *μ*g/mL DOE treatment further caused a dose-dependent reduction of the 80 nM VRI (vascular endothelial growth factor-VEGF receptor kinase inhibitor II) induced blood loss in SIVs area (embryos were coincubated with 80 nM VRI and 3 and 10 *μ*g/mL DOE from 24 to 72 hpf), while the same dependent dose of DOE caused a significant increase in VEGF receptors mRNAs expression, including* kdr*,* flt1*, and* kdr-like* from 24 to 72 hpf. Third, the proliferation of endothelial cells was a fundamental step of new blood capillary growth, and migration of endothelial cell was a critical event for angiogenesis; thereby, using MTT* in vitro *assay, 3–30 *μ*g/mL DOE also promoted the proliferation of HUVECs and markedly reduced the HUVECs injury induced by VRI for 24 h; utilizing the classical scratch model, DOE at 3 *μ*g/mL for 24 h significantly took part in promoting the migration of HUVECs. In other* in vitro *studies, the comprehensive results of an advanced molecular biology tool, named protein chip model, were used to investigate the mechanism of antibody-antigen interactions, which indicated that the* p*-Akt* Ser473* site was activated by 30 *μ*g/mL DOE for 24 h. Importantly, Akt activation based on the activity of PI3-kinase (PI3K) and VEGFR-2/PI3K/Akt pathway plays an important role in regulating the formation of new blood vessels and the survival of immature vessels [70, 71]. The phosphorylation of glycogen synthase kinase-3 (GSK-3) at* Ser*9 site depended on the activation of* p*-Akt, which might suppress the apoptosis of HUVECs [70, 72, 73]. Besides these, MAPK pathway could be activated by PI3K indirectly [70, 74]. Thus, the angiogenic effects of DOE were also understood through the activation of PI3K/MAPK pathways. Indeed, 30 *μ*M LY2940002 was shown to block the activation of* p*-Akt and* p*-GSK-3*β* induced by 30 *μ*g/mL DOE; meanwhile, activation of* p*-MEK 1/2 MAPK and* p*-Erk 1/2 MAPK induced by 3–30 *μ*g/mL DOE was also observed in the western blot analysis [70].

By screening of Chinese medicinal herbs on angiogenesis and antiangiogenesis activities, followed by using chick embryo chorioallantoic membrane (CAM) and bovine aortic endothelial cells (BAECs) models, Wang et al. (2004) suggested that the aqueous extract of* D. odorifera* root heartwood (core of root) strongly participated in angiogenesis activity with increased percentages of 95.24 ± 11.28% (0.2 g herb/mL) and 169.05 ± 30.28% (1 g herb/mL) in CAM model and 37.05 ± 3.39% (0.2 g herb/mL) and 50.35 ± 7.11% (1 g herb/mL) in BAECs model, as compared to those of the aqueous extract of the highest potential plant* Trichosanthes kirilowii* fruit wall [114.29 ± 22.39% (0.2 g herb/mL) and 183.81 ± 29.56% (1 g herb/mL) in CAM model and 56.47 ± 7.69% (0.2 g herb/mL) and 102.65 ± 11.52% (1 g herb/mL) in BAECs model] and positive control bFGF (basic fibroblast growth factor) [156.19 ± 28.55% (0.2 g herb/mL) and 211.43 ± 35.65% (1 g herb/mL) in CAM model and 161.39 ± 41.68% (0.2 g herb/mL) and 216.92 ± 19.57% (1 g herb/mL) in BAECs model]; however, this plant did not act on antiangiogenesis activity [69].

## 5. Conclusion

Taken together, the endemic plant* D. odorifera* has already been fully researched and is outlined in this paper. Based on technical chromatography and spectroscopic studies, phytochemical investigations of different parts (heartwood, root, root heartwood, and leaves) of the medicinal plant* D. odorifera* species led to the isolation of major flavonoids, phenols, and sesquiterpenes, as well as arylbenzofurans, quinones, and several fatty acid derivatives. Regarding the 91 different flavonoids described in the current review paper, an isoflavone formononetin** (4)**, two flavanones (2*S*)-liquiritigenin** (21)** and (2*S*)-pinocembrin** (24)**, an isoflavanone (3*R*)-sativanone** (35)**, a chalcone isoliquiritigenin** (65)**, and a pterocarpan medicarpin** (71)** are the most frequently found. Parallel with phytochemical analyses, biological experiments, such as cytotoxic assays; antibacterial, antioxidative, anti-inflammatory, antiosteosarcoma, antiosteoporosis, antiangiogenesis, and prostaglandin biosynthetic enzyme inhibition activities; vasorelaxant activities; and alpha-glucosidase inhibitory activities, suggested that the isolated compounds, along with the extracts, and fractions indicated the efficacious properties for drug development. Finally, extensive researches on* D. odorifera* and its other related species are expected.

## Figures and Tables

**Table 1:** Chemical constituents from *Dalbergia odorifera* species.

Number	Compounds	Parts	References
*Flavonoids*			

*Flavones and isoflavones*			
**1**	7-Methoxy-3,3′,4′,6-tetrahydroxyflavone	Heartwood	[18]
**2**	Fisetin	Heartwood	[18]
**3**	4′,5,7-Trihydroxy-3-methoxyflavone	Root	[19]
**4**	Formononetin	Heartwood Root heartwood Water fraction^*∗*^	[3, 18, 20–26], [27]^*∗*^
**5**	2′-*O*-Methylformononetin	Heartwood	[24]
**6**	Daidzein	Heartwood	[24]
**7**	3′-Hydroxydaidzein	Heartwood	[18, 20]
**8**	3′-Methoxydaidzein	Root	[19, 20]
**9**	2′,7-Dihydroxy-4′,5′-dimethoxyisoflavone	Heartwood	[18]
**10**	7,3′-Dihydroxy-5′-methoxyisoflavone	Heartwood	[28, 29]
**11**	Tectorigenin	Heartwood	[3, 23, 30]
**12**	Koparin	Heartwood	[18]
**13**	Xenognosin B	Heartwood Water fraction^*∗*^	[18], [27]^*∗*^
**14**	Prunetin	Leaves	[31]
**15**	Genistein	Leaves	[14, 31, 32]
**16**	Biochanin A	Leaves Heartwood	[14, 31–33]
**17**	Biochanin B	Heartwood Heartwood	[33]
**18**	Olibergin A	Heartwood	[24]
**19**	Orobol	Heartwood	[24]
**20**	Bowdichione	Heartwood	[18, 20]

*Flavanones, flavans, isoflavanones, and isoflavans*			
**21**	(2*S*)-Liquiritigenin	Heartwood Water fraction^*∗*^	[3, 18, 23, 29], [27]^*∗*^, [25]
**22**	Eriodictyol	Heartwood	[33]
**23**	Naringenin	Heartwood Water fraction	[27]^*∗*^, [33]
**24**	(2*S*)-Pinocembrin	Heartwood Water fraction^*∗*^	[24, 29], [27]^*∗*^, [33]
**25**	3′,4′,7-Trihydroxyflavanone	Heartwood Water fraction^*∗*^	[18, 34], [27]^*∗*^
**26**	(2*S*)-Pinostrobin	Heartwood	[24]
**27**	(2*S*)-3′,5,5′,7-Tetrahydroxyflavanone	Heartwood	[24]
**28**	(2*S*)-7-Methoxy-4′,6-dihydroxyflavanone	Heartwood	[24]
**29**	Carthamidin	Heartwood	[34]
**30**	6,7,4′-Trihydroxyflavanone	Heartwood	[28]
**31**	6,4′-Dihydroxy-7-methoxyflavanone	Heartwood	[28]
**32**	(2*S*)-6,7,4′-Trihydroxyflavan	Heartwood	[28]
**33**	(2*S*)-6,4′-Dihydroxy-7-methoxyflavan	Heartwood	[28, 29]
**34**	6,7-Dimethoxy-2-(4-methoxybenzoquinonyl)flavan	Heartwood	[35]
**35**	(3*R*)-Sativanone	Heartwood	[18, 24, 29, 33, 34]
**36**	(3*R*)-Violanone	Heartwood Water fraction	[24], [27]^*∗*^
**37**	(3*R*)-3′-*O*-Methylviolanone	Heartwood Water fraction^*∗*^	[18, 24, 29], [27]^*∗*^
**38**	(3*R*)-Vestitone	Heartwood Water fraction^*∗*^	[34], [27]^*∗*^
**39**	(3*S*)-2′,4′,5′-Trimethoxy-7-hydroxyisoflavanone	Heartwood	[24]
**40**	(3*R*)-2′,3′,7-Trihydroxy-4′-methoxyisoflavanone	Heartwood Root	[19, 20, 34]
**41**	(3*R*)-4′-Methoxy-2′,3,7-trihydroxyisoflavanone	Heartwood Water fraction^*∗*^	[18, 24, 34], [27]^*∗*^
**42**	(3*S*)-2′,4′-Dimethoxy-3,7-dihydroxyisoflavanone	Heartwood	[24]
**43**	(3*R*)-7,3′-Dihydroxy-6,2′,4′-trimethoxyisoflavanone	Heartwood	[7]
**44**	(3*R*)-Vestitol	Heartwood Root heartwood Root	[19–22, 34]
**45**	(3*R*)-5′-Methoxyvestitol	Heartwood Root	[3, 20, 23, 25]
**46**	(3*R*)-3′,8-Dihydroxyvestitol	Heartwood	[20]
**47**	Duratin	Heartwood Root heartwood	[21, 22]
**48**	Isoduratin	Heartwood Root heartwood	[21, 22]
**49**	Mucronulatol	Heartwood Root heartwood	[3, 21–23]
**50**	(3*R*)-Calussequinone	Heartwood	[3, 20, 23, 26]
**51**	7-Hydroxy-5′-methoxyspiro[benzo[d][1,3]dioxole-2′,3-chroman]-4-one	Heartwood	[35]
**52**	Odoriflavene	Heartwood Root heartwood Root	[21, 22, 25]

*Neoflavones*			
**53**	Dalbergin	Heartwood	[18, 28, 36]
**54**	Stevenin	Heartwood	[18, 36]
**55**	Melanettin	Heartwood	[18, 24, 36]
**56**	3′-Hydroxymelanettin	Heartwood	[18, 24, 33, 36]
**57**	*R*(−)-Latifolin	Heartwood	[24, 28, 29, 37]
**58**	*R*(−)-5-*O*-Methylatifolin	Heartwood	[24, 28]
**59**	*R*(−)-Dalbergiphenol	Heartwood	[28]
**60**	9-Hydroxy-6,7-dimethoxydalbergiquinol	Heartwood	[28]
**61**	4,5-Dimethoxy-2-hydroxydalbergiquinol	Heartwood	[24]
**62**	2,4,5-Trimethoxy-3′-hydroxydalbergiquinol	Heartwood	[24]
**63**	2,4,5-Trimethoxydalbergiquinol	Heartwood	[38]
**64**	3′-Hydroxy-2,4,5-trimethoxydalbergiquinol	Heartwood	[36]

*Chalcones*			
**65**	Isoliquiritigenin	Heartwood	[18, 24, 28, 29, 34]
**66**	2′-*O*-Methyl-isoliquiritigenin	Heartwood Root	[20, 25]
**67**	4,2′,5′-Trihydroxy-4′-methoxychalcone	Heartwood	[28]
**68**	Butein	Heartwood	[18, 24]
**69**	*α*,2′,3,4,4′-Pentahydroxydihydrochalcone	Heartwood	[24]
**70**	*α*,2′,4,4′-Tetrahydroxydihydrochalcone	Heartwood	[24]

*Pterocarpans*			
**71**	Medicarpin	Heartwood Root heartwood Root CHCl_3_ extract^*∗∗*^	[2, 18–24, 26], [39]^*∗∗*^
**72**	(6a*R*,11a*R*)-6a,9-Dimethoxy-3-hydroxypterocarpan	Heartwood	[7]
**73**	(6a*R*,11a*R*)-6a,3,9-Trimethoxypterocarpan	Heartwood	[7]
**74**	(6a*R*,11a*R*)-Variabiin	Heartwood	[7]
**75**	Vesticarpan	Heartwood	[7]
**76**	3-Methoxy-9-hydroxypterocarpan	Heartwood	[7]
**77**	Meliotocarpan A	Heartwood	[7, 18]
**78**	Meliotocarpan C	Heartwood Root heartwood	[21, 22]
**79**	Meliotocarpan D	Heartwood Root heartwood	[7, 21, 22]
**80**	Methylnissolin	Heartwood Root heartwood	[21, 22]
**81**	Odoricarpan	Heartwood Root heartwood	[21, 22]
**82**	3-Hydroxy-9-methoxycoumestan	Heartwood	[18]

*Bisflavonoids*			
**83**	(3*R*,4*R*)-*trans*-2′,3′,7-Trihydroxy-4′-methoxy-4-[(3*R*)-2′,7-dihydroxy-4′-methoxyisoflavan-5′-yl]isoflavan	Heartwood	[20]
**84**	(3*R*,4*R*)-*trans*-2′,7-Dihydroxy-4′-methoxy-4-[(3*R*)-2′,7-dihydroxy-4′-methoxyisoflavan-5′-yl]isoflavan	Heartwood	[20]
**85**	(3*R*,4*R*)-*trans*-2′,7-Dihydroxy-4′,5′-dimethoxy-4-[(3*R*)-2′,7-dihydroxy-4′-methoxyisoflavan-5′-yl]isoflavan	Heartwood	[20]
**86**	(3*R*,4*R*)-*trans*-3′,7-Dihydroxy-2′,5′-dimethoxy-4-[(3*R*)-2′,7-dihydroxy-4′-methoxyisoflavan-5′-yl]isoflavan	Heartwood	[20]
**87**	(3*R*,4*R*)-*trans*-3′,7-Dihydroxy-2′,5′-dimethoxy-4-[(3*R*)-2′,7-dihydroxy-4′-methoxyisoflavan-5′-yl]isoflavan	Heartwood	[40]
**88**	DO-17^*∗*^	Heartwood	[20]
**89**	DO-19^*∗*^	Heartwood	[40]
**90**	DO-20^*∗*^	Heartwood	[40]
**91**	DO-21^*∗*^	Heartwood	[40]

*Phenols*			

**92**	2-(2-(2,4-Dimethoxyphenyl)-2-oxoethoxy)-4-hydrobenzoic acid	Heartwood	[7]
**93**	2-(2,4-Dihydroxyphenyl)-1-(4-hydroxy-2-methoxyphenyl) ethanone	Heartwood	[7]
**94**	Obtustyrene	Heartwood Root heartwood	[21, 22]
**95**	Hydroxyobtustyrene	Heartwood Root heartwood	[21–23]
**96**	Isomucronustyrene	Heartwood Root heartwood	[21, 22]
**97**	Cearoin	Heartwood	[18, 28, 36]
**98**	2,2′,5-Trihydroxy-4-methoxybenzophenone	Heartwood	[28]
**99**	2,4-Dihydroxy-5-methoxybenzophenone	Root	[19]
**100**	Methyl-2-hydroxy-3,4-dimethoxybenzoate	Heartwood Root heartwood	[21, 22]
**101**	2-Hydroxy-3,4-dimethoxybenzaldehyde	Heartwood	[23]

*Sesquiterpenes*			

**102**	6*α*-Hydroxycyclonerolidol	Heartwood	[41]
**103**	*Rel*-(3*R*,6*R*,7*S*)-3,7,11-Trimethyl-3,7-epoxy-1,10-dodecadien-6-ol	Heartwood	[41]
**104**	*Rel*-(3*S*,6*R*,7*S*,10*S*)-2,6,10-Trimethyl-3,6,7,10-diepoxy-2-dodecen-11-ol	Heartwood	[41]
**105**	*Rel*-(3*S*,6*R*,7*S*,10*R*)-2,6,10-Trimethyl-3,6,7,10-diepoxy-11-dodecen-2-ol	Heartwood	[41]
**106**	*Rel*-(2*R*,2′*R*,5′*S*)-2,5′-Dimethyl-5′-vinylhexahydro-2,2′-bifuran-5(2*H*)-one	Heartwood	[41]
**107**	Crocinervolide	Heartwood	[41]
**108**	Neroplofurol	Heartwood	[41]
**109**	*Rel*-(3*S*,6*R*,7*S*,9*E*)-3,7,11-Trimethyl-3,6-epoxy-1,9,11-dodecatrien-7-ol	Heartwood	[41]
**110**	*Rel*-(3*S*,6*R*,7*S*)-3,7,11-Trimethyl-3,6-epoxy-1-dodecen-7,11-diol	Heartwood	[41]
**111**	*Rel*-(3*S*,6*R*,7*S*)-3,7,11-Trimethyl-3,6-epoxy-1,10-dodecadien-7-ol	Heartwood	[41]
**112**	*Rel*-(3*S*,6*S*,7*R*)-3,7,11-Trimethyl-3,6-epoxy-1,10-dodecadien-7-ol	Heartwood	[41]
**113**	(3*S*,6*R*,7*R*)-3,7,11-Trimethyl-3,6-epoxy-1,10-dodecadien-7-ol	Heartwood	[42]
**114**	(3*S*,6*S*,7*R*)-3,7,11-Trimethyl-3,6-epoxy-1,10-dodecadien-7-ol	Heartwood	[42]
**115**	(*E*)-7-Hydroxy-6,10-dimethylundeca-5,9-dien-2-one	Heartwood	[41]
**116**	3,7,11-Trimethyldodeca-1,10-diene-3,6,7-triol	Heartwood	[41]
**117**	(3*S*,5*E*)-3,11-Dimethyl-7-methylenedodaca-1,5,10-trien-3-ol	Heartwood	[41]
**118**	*Rel*-(*S*,*E*)-2-[(S)-2,2-Dimethyl-1,3-dioxolan-4-yl]-6,10-dimethylundeca-5,9-dien-2-ol	Heartwood	[41]

*Arylbenzofurans*			

**119**	(2*R*, 3*R*)-Obtusafuran	Heartwood	[4, 28]
**120**	6-Methoxy-5,2′,4′-trihydroxy-3-benzoylbenzofuran	Heartwood	[7]
**121**	Phenylbenzofuran I	Heartwood	[35]
**122**	Sulfuretin	Heartwood	[34]
**123**	Isoparvifuran	Heartwood	[28]
**124**	2′,6-Dihydroxy-4′-methoxy-2-arylbenzofuran (6-hydroxy-2-(2-hydroxy-4-methoxyphenyl)benzofuran)	Heartwood CHCl_3_ extract^*∗∗*^	[40], [39]^*∗∗*^

*Quinones*			

**125**	(*S*)-4-Methoxydalbergione	Heartwood	[18]
**126**	*R*(+)-4-Methoxydalbergione	Heartwood	[28, 36]
**127**	4′-Hydroxy-4-methoxydalbergione	Heartwood	[28]

*Other components*			

**128**	2-Methoxy-3-hydroxyxanthone	Heartwood	[24]
**129**	Hexanoic acid, 2-propenyl ester	Root	[19]
**130**	Hexadecanoic acid, ethyl ester	Root	[19]
**131**	3,8-Nonadien-2-one	Root	[19]

^*∗*^Not to show part use. ^*∗∗*^Not name.

**Table 2:** Biological experiments from isolated constituents from *Dalbergia odorifera* species.

Number	Compounds	Biological experiments	References
**3**	4′,5,7-Trihydroxy-3-methoxyflavone	Antioxidant	[19]
**4**	Formononetin	Alpha-glucoside inhibition, antibacterial, antioxidant, anti-inflammatory, cytotoxicity	[3, 23–26]
**5**	2′-*O*-Methylformononetin	Anti-inflammatory	[24]
**6**	Daidzein	Anti-inflammatory	[24]
**8**	3′-Methoxydaidzein	Antioxidant	[19]
**10**	7,3′-Dihydroxy-5′-methoxyisoflavone	Alpha-glucoside inhibition, anti-inflammatory	[28, 29]
**11**	Tectorigenin	Alpha-glucoside inhibition, cytotoxicity	[3, 23]
**12**	Koparin	Anti-inflammatory	[18]
**13**	Xenognosin B	Anti-inflammatory	[18]
**16**	Biochanin A	Antioxidant	[33]
**17**	Biochanin B	Antioxidant	[33]
**18**	Olibergin A	Anti-inflammatory	[24]
**19**	Orobol	Anti-inflammatory	[24]
**20**	Bowdichione	Anti-inflammatory	[18]
**21**	(2*S*)-Liquiritigenin	Alpha-glucoside inhibition, antibacterial, cytotoxicity	[3, 23, 29, 34]
**22**	Eriodictyol	Antioxidant	[33]
**23**	Naringenin	Antioxidant	[33]
**24**	(2*S*)-Pinocembrin	Alpha-glucoside inhibition, antioxidant, anti-inflammatory	[24, 29, 33]
**26**	(2*S*)-Pinostrobin	Anti-inflammatory	[24]
**27**	(2*S*)-3′,5,5′,7-Tetrahydroxyflavanone	Anti-inflammatory	[24]
**28**	(2*S*)-7-Methoxy-4′,6-dihydroxyflavanone	Anti-inflammatory	[24]
**29**	Carthamidin	Antibacterial	[34]
**31**	6,4′-Dihydroxy-7-methoxyflavanone	Anti-inflammatory	[28, 43]
**32**	(2*S*)-6,7,4′-Trihydroxyflavan	Anti-inflammatory	[28]
**33**	(2*S*)-6,4′-Dihydroxy-7-methoxyflavan	Alpha-glucoside inhibition, anti-inflammatory	[28, 29]
**34**	6,7-Dimethoxy-2-(4-methoxybenzoquinonyl)-flavan	Cytotoxicity	[35]
**35**	(3*R*)-Sativanone	Alpha-glucoside inhibition, antibacterial, antioxidant, anti-inflammatory	[24, 29, 33, 34]
**36**	(3*R*)-Violanone	Anti-inflammatory	[24]
**37**	(3*R*)-3′-*O*-Methylviolanone	Anti-inflammatory	[18, 24]
**38**	(3*R*)-Vestitone	Antibacterial	[34]
**39**	(3*S*)-2′,4′,5′-Trimethoxy-7-hydroxyisoflavanone	Anti-inflammatory	[24]
**40**	(3*R*)-2′,3′,7-Trihydroxy-4′-methoxyisoflavanone	Antioxidant, antibacterial	[19, 20, 34]
**41**	(3*R*)-4′-Methoxy-2′,3,7-trihydroxyisoflavanone	Anti-inflammatory, antibacterial	[24, 34]
**42**	(3*S*)-2′,4′-Dimethoxy-3,7-dihydroxyisoflavanone	Anti-inflammatory	[24]
**43**	(3*R*)-7,3′-Dihydroxy-6,2′,4′-trimethoxyisoflavanone	Antibacterial, cytotoxicity	[7]
**44**	(3*R*)-Vestitol	Antioxidant, antibacterial, cytotoxicity, PG synthetase inhibition	[19, 21, 22, 34]
**45**	(3*R*)-5′-Methoxyvestitol	Alpha-glucoside inhibition, antioxidant, cytotoxicity	[3, 23, 25]
**48**	Isoduratin	PG synthetase inhibition	[21, 22]
**49**	Mucronulatol	Alpha-glucoside inhibition, cytotoxicity, PG synthetase inhibition	[3, 21–23]
**50**	(3*R*)-Calussequinone	Alpha-glucoside inhibition, antibacterial, cytotoxicity	[3, 23, 26]
**51**	7-Hydroxy-5′-methoxyspiro[benzo[d][1,3]dioxole-2′,3-chroman]-4-one	Cytotoxicity	[35]
**52**	Odoriflavene	Antioxidant, cytotoxicity, PG synthetase inhibition	[21, 22, 25]
**55**	Melanettin	Anti-inflammatory	[24]
**56**	3′-Hydroxymelanettin	Antioxidant, anti-inflammatory	[24, 33]
**57**	*R*(−)-Latifolin	Alpha-glucoside inhibition, anti-inflammatory	[24, 28, 29, 37]
**58**	*R*(−)-5-*O*-Methylatifolin	Anti-inflammatory	[28]
**59**	*R*(−)-Dalbergiphenol	Anti-inflammatory	[28]
**60**	9-Hydroxy-6,7-dimethoxydalbergiquinol	Anti-inflammatory	[5, 28]
**61**	4,5-Dimethoxy-2-hydroxydalbergiquinol	Anti-inflammatory	[24]
**62**	2,4,5-Trimethoxy-3′-hydroxydalbergiquinol	Anti-inflammatory	[24]
**65**	Isoliquiritigenin	Alpha-glucoside inhibition, antibacterial, anti-inflammatory	[18, 24, 28, 29, 34, 44]
**66**	2′-*O*-Methyl-isoliquiritigenin	Antioxidant, cytotoxicity	[25]
**67**	4,2′,5′-Trihydroxy-4′-methoxychalcone	Anti-inflammatory	[28, 45]
**68**	Butein	Anti-inflammatory, vasorelaxant activity	[24, 46, 47]
**69**	*α*,2′,3,4,4′-Pentahydroxydihydrochalcone	Anti-inflammatory	[24]
**70**	*α*,2′,4,4′-Tetrahydroxydihydrochalcone	Anti-inflammatory	[24]
**71**	Medicarpin	Alpha-glucoside inhibition, antibacterial, antioxidant, anti-inflammatory, cytotoxicity	[3, 19, 23, 26], [39]^*∗∗*^
**72**	(6a*R*,11a*R*)-6a,9-Dimethoxy-3-hydroxypterocarpan	Cytotoxicity	[7]
**73**	(6a*R*,11a*R*)-6a,3,9-Trimethoxypterocarpan	Antibacterial, cytotoxicity	[7]
**74**	(6a*R*,11a*R*)-Variabiin	Antibacterial, cytotoxicity	[7]
**75**	Vesticarpan	Cytotoxicity	[7]
**76**	3-Methoxy-9-hydroxypterocarpan	Antibacterial, cytotoxicity	[7]
**77**	Meliotocarpan A	Antibacterial, cytotoxicity	[7]
**79**	Meliotocarpan D	Antibacterial, cytotoxicity	[7]
**92**	2-(2-(2,4-Dimethoxyphenyl)-2-oxoethoxy)-4-hydrobenzoic acid	Antibacterial, cytotoxicity	[7]
**93**	2-(2,4-Dihydroxyphenyl)-1-(4-hydroxy-2-methoxyphenyl)ethanone	Cytotoxicity	[7]
**94**	Obtustyrene	PG synthetase inhibition	[21, 22]
**95**	Hydroxyobtustyrene	Cytotoxicity, PG synthetase inhibition	[21–23]
**96**	Isomucronustyrene	PG synthetase inhibition	[21, 22]
**97**	Cearoin	Anti-inflammatory	[18, 28]
**98**	2,2′,5-Trihydroxy-4-methoxybenzophenone	Anti-inflammatory	[28]
**99**	2,4-Dihydroxy-5-methoxybenzophenone	Antioxidant	[19]
**100**	Methyl-2-hydroxy-3,4-dimethoxybenzoate	PG synthetase inhibition	[21, 22]
**101**	2-Hydroxy-3,4-dimethoxybenzaldehyde	Cytotoxicity	[23]
**102**	6*α*-Hydroxycyclonerolidol	Antibacterial	[41]
**103**	*Rel*-(3*R*,6*R*,7*S*)-3,7,11-Trimethyl-3,7-epoxy-1,10-dodecadien-6-ol	Antibacterial	[41]
**104**	*Rel*-(3*S*,6*R*,7*S*,10*S*)-2,6,10-Trimethyl-3,6,7,10-diepoxy-2-dodecen-11-ol	Antibacterial	[41]
**105**	*Rel*-(3*S*,6*R*,7*S*,10*R*)-2,6,10-Trimethyl-3,6,7,10-diepoxy-11-dodecen-2-ol	Antibacterial	[41]
**106**	*Rel*-(2*R*,2′*R*,5′*S*)-2,5′-Dimethyl-5′-vinylhexahydro-2,2′-bifuran-5(2*H*)-one	Antibacterial	[41]
**107**	Crocinervolide	Antibacterial	[41]
**108**	Neroplofurol	Antibacterial	[41]
**109**	*Rel*-(3*S*,6*R*,7*S*,9*E*)-3,7,11-Trimethyl-3,6-epoxy-1,9,11-dodecatrien-7-ol	Antibacterial	[41]
**110**	*Rel*-(3*S*,6*R*,7*S*)-3,7,11-Trimethyl-3,6-epoxy-1-dodecen-7,11-diol	Antibacterial	[41]
**111**	*Rel*-(3*S*,6*R*,7*S*)-3,7,11-Trimethyl-3,6-epoxy-1,10-dodecadien-7-ol	Antibacterial	[41]
**112**	*Rel*-(3*S*,6*S*,7*R*)-3,7,11-Trimethyl-3,6-epoxy-1,10-dodecadien-7-ol	Antibacterial	[41]
**113**	(3*S*,6*R*,7*R*)-3,7,11-Trimethyl-3,6-epoxy-1,10-dodecadien-7-ol	Antithrombotics, antiplatelet	[42]
**114**	(3*S*,6*S*,7*R*)-3,7,11-Trimethyl-3,6-epoxy-1,10-dodecadien-7-ol	Antithrombotics, antiplatelet	[42]
**115**	(*E*)-7-Hydroxy-6,10-dimethylundeca-5,9-dien-2-one	Antibacterial	[41]
**116**	3,7,11-Trimethyldodeca-1,10-diene-3,6,7-triol	Antibacterial	[41]
**117**	(3*S*,5*E*)-3,11-Dimethyl-7-methylenedodaca-1,5,10-trien-3-ol	Antibacterial	[41]
**118**	*Rel*-(*S*,*E*)-2-[(S)-2,2-Dimethyl-1,3-dioxolan-4-yl]-6,10-dimethylundeca-5,9-dien-2-ol	Antibacterial	[41]
**119**	(2*R*,3*R*)-Obtusafuran	Anti-inflammatory	[4, 28]
**120**	6-Methoxy-5,2′,4′-trihydroxy-3-benzoylbenzofuran	Antibacterial, cytotoxicity	[7]
**121**	Phenylbenzofuran I	Cytotoxicity	[35]
**122**	Sulfuretin	Antibacterial	[34]
**123**	Isoparvifuran	Anti-inflammatory	[4, 28]
**124**	2′,6-Dihydroxy-4′-methoxy-2-arylbenzofuran (6-hydroxy-2-(2-hydroxy-4-methoxyphenyl)benzofuran)	Anti-inflammatory	[39]
**125**	(*S*)-4-Methoxydalbergione	Anti-inflammatory, antiosteosarcoma	[18, 48]
**126**	*R*(+)-4-Methoxydalbergione	Anti-inflammatory, antiosteosarcoma	[28, 38]
**127**	4′-Hydroxy-4-methoxydalbergione	Anti-inflammatory	[28]
**128**	2-Methoxy-3-hydroxyxanthone	Anti-inflammatory	[24]
**129**	Hexanoic acid, 2-propenyl ester	Antioxidant	[19]
**130**	Hexadecanoic acid, ethyl ester	Antioxidant	[19]
**131**	3,8-Nonadien-2-one	Antioxidant	[19]

## Abbreviations

ITS: Internal transcribed spacer
TLC: Thin-layer chromatography
CC: Column chromatography
GC: Gas chromatography
HPLC: High-performance liquid chromatography
UPLC: Ultraperformance liquid chromatography
NMR: Nuclear magnetic resonance
MS: Mass spectrum
DES-NPCE: Deep eutectic solvent-based negative pressure cavitation assisted extraction
MA-ATPE: Microwave-assisted aqueous two-phase extraction
DM: Dry material
MAE: Microwave-assisted extraction
HRE: Heat reflux extraction
USE: Ultrasound-assisted extraction
EE: Ethyl acetate extract
BE: *n*-Butanol extract
PE: Petroleum extract
WE: Water extract
SDE: Simultaneous distillation and extraction
DPPH: 1,1-Diphenyl-2-picrylhydrazyl
BHT: Butylated hydroxytoluene
ABTS^•+^: 2,2-Azinobis(3-ethylbenzothiazoline-6-sulfonate) radical
OSI: Oil stability index
Pf: Protection factor
GSH: Glutathione
AAPH: 2,2-Azobis(2-amidinopropane dihydrochloride)
LDL: Low-density lipoprotein
NF-*κ*B: Nuclear factor-*κ*B
NO: Nitric oxide
TNF-*α*: Tumor necrosis factor-*α*
IL-1*β*: Interleukin-1*β*
iNOS: Nitric oxide synthase
COX-2: Cyclooxygenase-2
LPS: Lipopolysaccharide
HO-1: Hemeoxygenase-1
PGE_2_: Prostaglandin E2
SnPP: Tin protoporphyrin
MTT: 3-(4,5-Dimethylthiazol-2-yl)-2,5-diphenyl tetrazolium bromide salt
LTs: Leukotrienes
PMA: Phorbol myristate acetate
APTT: Activated partial thromboplastin time
TT: Thrombin time
PT: Prothrombin time
TXA2: Thromboxane A2
cAMP: Cyclic adenosine monophosphate
cGMP: Cyclic guanosine monophosphate
CREB: cAMP response element binding
PTEN: Protein phosphatase and tensin homolog deleted on chromosome ten
PCNA: Proliferating cell nuclear antigen
ALP: Alkaline phosphatase activity
BSP: Bone sialoprotein
OPN: Osteopontin
OCN: Osteocalcin
BMP: Bone morphogenetic protein
DKK1: Dickkopf-1
PDEs: Phosphodiesterases
L-NMMA: N^G^-monomethyl-L-arginine
DEAE: Diethylaminoethyl
EDTA: Ethylenediaminetetraacetic acid
Vit. C: Vitamin C
PG: Prostaglandin
DOE: * D. odorifera* heartwood
HMGB1: High mobility group box 1
ECM: Extracellular matrix
TGF-*β*1: Transforming growth factor-*β*1
TIMP: Tissue inhibitor of metalloproteinase
HUVEC: Human umbilical vein endothelial cell
PI3K: PI3-kinase
CAM: Chorioallantoic membrane
BAECs: Bovine aortic endothelial cells
bFGF: Basic fibroblast growth factor.

## References

[1] Zheng L. W. L., Guo X., Li X., Chen Z. (2011). Essential oil composition from the seeds of *Dalbergia odorifera* T. Chen grown in Hainan, China. *Journal of Food, Agriculture & Environment*.

[2] Sun S., Zeng X., Zhang D., Guo S. (2015). Diverse fungi associated with partial irregular heartwood of Dalbergia odorifera. *Scientific Reports*.

[3] Choi C. W., Choi Y. H., Cha M.-R. (2010). Yeast *α*-glucosidase inhibition by isoflavones from plants of leguminosae as an in vitro alternative to acarbose. *Journal of Agricultural and Food Chemistry*.

[4] Lee D.-S., Jeong G.-S. (2014). Arylbenzofuran isolated from Dalbergia odorifera suppresses lipopolysaccharide-induced mouse BV2 microglial cell activation, which protects mouse hippocampal HT22 cells death from neuroinflammation-mediated toxicity. *European Journal of Pharmacology*.

[5] Lee D.-S., Li B., Keo S. (2013). Inhibitory effect of 9-hydroxy-6,7-dimethoxydalbergiquinol from Dalbergia odorifera on the NF-*κ*B-related neuroinflammatory response in lipopolysaccharide-stimulated mouse BV2 microglial cells is mediated by heme oxygenase-1. *International Immunopharmacology*.

[6] Li X., Wu L., Liu W. (2014). A network pharmacology study of Chinese medicine QiShenYiQi to reveal its underlying multi-compound, multi-target, multi-pathway mode of action. *PLoS ONE*.

[7] Wang H., Mei W.-L., Zeng Y.-B. (2014). Phenolic compounds from Dalbergia odorifera. *Phytochemistry Letters*.

[8] Zheng X., Zhao X., Wang S., Luo K., Wei Y., Zheng J. (2007). Co-administration of Dalbergia odorifera increased bioavailability of Salvia miltiorrhizae in rabbits. *American Journal of Chinese Medicine*.

[9] Li B., Xu X., Wang X. (2012). A systems biology approach to understanding the mechanisms of action of Chinese herbs for treatment of cardiovascular disease. *International Journal of Molecular Sciences*.

[10] Chen Y., Li Q., Pan C. (2015). QiShenYiQi Pills, a compound in Chinese medicine, protects against pressure overload-induced cardiac hypertrophy through a multi-component and multi-target mode. *Scientific Reports*.

[11] Sugiyama A., Zhu B.-M., Takahara A., Satoh Y., Hashimoto K. (2002). Cardiac effects of Salvia miltiorrhiza/Dalbergia odorifera mixture, an intravenously applicable Chinese medicine widely used for patients with ischemic heart disease in China. *Circulation Journal*.

[12] Saha S., Mondal H., Hossain F., Anisuzzman M., Hasan M. M., Cordell G. A. (2013). Ethnomedicinal, phytochemical, and pharmacological profile of the genus Dalbergia L. (Fabaceae). *Phytopharmacology*.

[13] Dalbergia http://www.theplantlist.org/search/.

[14] Ma F.-Y., Gu C.-B., Li C.-Y. (2013). Microwave-assisted aqueous two-phase extraction of isoflavonoids from Dalbergia odorifera T. Chen leaves. *Separation and Purification Technology*.

[15] Hao B.-Z., Wu J.-L. (1993). Vacuole proteins in parenchyma cells of secondary phloem and xylem of Dalbergia odorifera. *Trees - Structure and Function*.

[16] Liu X., Xu D., Yang Z., Zhang N. (2017). Geographic variations in seed germination of Dalbergia odorifera T. Chen in response to temperature. *Industrial Crops and Products*.

[17] Lu J. K., He X. H., Huang L. B., Kang L. H., Xu D. P. (2012). Two Burkholderia strains from nodules of Dalbergia odorifera T. Chen in Hainan Island, southern China. *New Forests*.

[18] Chan S.-C., Chang Y.-S., Wang J.-P., Chen S.-C., Kuo S.-C. (1998). Three new flavonoids and antiallergic, anti-inflammatory constituents from the heartwood of *Dalbergia odorifera*. *Planta Medica*.

[19] Wang W., Weng X., Cheng D. (2000). Antioxidant activities of natural phenolic components from *Dalbergia odorifera T. Chen*. *Food Chemistry*.

[20] Yahara S., Ogata T., Nohara N. (1989). Isoflavan and Related Compounds from Dalbergia odorifera. I. *Chemical & Pharmaceutical Bulletin*.

[21] Goda Y., Katayama M., Ichikawa K., Shibuya M., Kiuchi F., Sankawa U. (1985). Inhibitors of prostaglandin biosynthesis from dalbergia odorifera. *Chemical & Pharmaceutical Bulletin*.

[22] Goda Y., Kiuchi F., Shibuya M., Sankawa U. (1992). Inhibitors of prostaglandin biosynthesis from *Dalbergia odorifera*. *Chemical & Pharmaceutical Bulletin*.

[23] Choi C. W., Choi Y. H., Cha M. R. (2009). Antitumor components isolated from the heartwood extract of *Dalbergia odorifera*. *Journal of The Korean Society for Applied Biological Chemistry*.

[24] Lee C., Lee J. W., Jin Q. (2013). Inhibitory constituents of the heartwood of Dalbergia odorifera on nitric oxide production in RAW 264.7 macrophages. *Bioorganic & Medicinal Chemistry Letters*.

[25] Yu X., Wang W., Yang M. (2007). Antioxidant activities of compounds isolated from Dalbergia odorifera T. Chen and their inhibition effects on the decrease of glutathione level of rat lens induced by UV irradiation. *Food Chemistry*.

[26] Islam T. (2008). Secondary Metabolites from Nonhost Plants Affect the Motility and Viability of Phytopathogenic Aphanomyces cochlioides Zoospores. *Zeitschrift für Naturforschung C*.

[27] Feng J., Xiao Y., Guo Z., Yu D., Jin Y., Liang X. (2011). Purification of compounds from Lignum Dalbergia Odorifera using two-dimensional preparative chromatography with Click oligo (ethylene glycol) and C18 column. *Journal of Separation Science*.

[28] An R.-B., Jeong G.-S., Kim Y.-C. (2008). Flavonoids from the heartwood of Dalbergia odorifera and their protective effect on glutamate-induced oxidative injury in HT22 cells. *Chemical & Pharmaceutical Bulletin*.

[29] Zhao C., Liu Y., Cong D. (2013). Screening and determination for potential *α*-glucosidase inhibitory constituents from Dalbergia odorifera T. Chen using ultrafiltration-LC/ESI-MSn. *Biomedical Chromatography*.

[30] Xu L., Shi H., Liang T. (2011). Selective separation of flavonoid glycosides in *Dalbergia odorifera* by matrix solid-phase dispersion using titania. *Journal of Separation Science*.

[31] Li L., Liu J.-Z., Luo M. (2016). Efficient extraction and preparative separation of four main isoflavonoids from Dalbergia odorifera T. Chen leaves by deep eutectic solvents-based negative pressure cavitation extraction followed by macroporous resin column chromatography. *Journal of Chromatography B*.

[32] Ma F.-Y., Luo M., Zhao C.-J. (2013). Simple and efficient preparation of biochanin A and genistein from Dalbergia odorifera T. Chen leaves using macroporous resin followed by flash chromatography. *Separation and Purification Technology*.

[33] Hou J.-P., Wu H., Ho C.-T., Weng X.-C. (2011). Antioxidant activity of polyphenolic compounds from Dalbergia odorifera T. Chen. *Pakistan Journal of Nutrition*.

[34] Zhao X., Mei W., Gong M., Zuo W., Bai H., Dai H. (2011). Antibacterial activity of the flavonoids from Dalbergia odorifera on Ralstonia solanacearum. *Molecules*.

[35] Wang H., Dong W.-H., Zuo W.-J. (2014). Three new phenolic compounds from Dalbergia odorifera. *Journal of Asian Natural Products Research*.

[36] Chan S.-C., Chang Y.-S., Kuo S.-C. (1997). Neoflavonoids from *Dalbergia odorifera*. *Phytochemistry*.

[37] Lee D.-S., Kim K.-S., Ko W. (2014). The neoflavonoid latifolin isolated from meoh extract of Dalbergia odorifera attenuates inflammatory responses by inhibiting NF-*κ*B activation via Nrf2-mediated heme oxygenase-1 expression. *Phytotherapy Research*.

[38] Yun H.-M., Park K.-R., Quang T. H. (2015). 2,4,5-Trimethoxyldalbergiquinol promotes osteoblastic differentiation and mineralization via the BMP and Wnt/*β*-catenin pathway. *Cell Death & Disease*.

[39] Miller D. K., Sadowski S., Han G. Q., Joshua H. (1989). Identification and isolation of medicarpin and a substituted benzofuran as potent leukotriene inhibitors in an anti-inflammatory Chinese herb. *Prostaglandins, Leukotrienes and Essential Fatty Acids*.

[40] Ogata T., Yahara S., Hisatsune R., Konishi R., Nohara T. (1990). Isoflavan and related compounds from *Dalbergia odorifera*. *Chemical and Pharmceutical Bulletin*.

[41] Wang H., Dong W.-H., Zuo W.-J. (2014). Five new sesquiterpenoids from *Dalbergia odorifera*. *Fitoterapia*.

[42] Tao Y., Wang Y. (2010). Bioactive sesquiterpenes isolated from the essential oil of *Dalbergia odorifer*a T. Chen. *Fitoterapia*.

[43] Li B., Lee D.-S., Jeong G.-S., Kim Y.-C. (2012). Involvement of heme oxygenase-1 induction in the cytoprotective and immunomodulatory activities of 6,4′-dihydroxy-7-methoxyflavanone in murine hippocampal and microglia cells. *European Journal of Pharmacology*.

[44] Lee S. H., Kim J. Y., Seo G. S., Kim Y.-C., Sohn D. H. (2009). Isoliquiritigenin, from *Dalbergia odorifera*, up-regulates anti-inflammatory heme oxygenase-1 expression in RAW264.7 macrophages. *Inflammation Research*.

[45] Lee D.-S., Li B., Im N.-K., Kim Y.-C., Jeong G.-S. (2013). 4,2′,5′-Trihydroxy-4′-methoxychalcone from dalbergia odorifera exhibits anti-inflammatory properties by inducing heme oxygenase-1 in murine macrophages. *International Immunopharmacology*.

[46] Cheng Z.-J., Kuo S.-C., Chan S.-C., Ko F.-N., Teng C.-M. (1998). Antioxidant properties of butein isolated from *Dalbergia odorifera*. *Biochimica et Biophysica Acta*.

[47] Yu S.-M., Cheng Z.-J., Kuo S.-C. (1995). Endothelium-dependent relaxation of rat aorta by butein, a novel cyclic AMP-specific phosphodiesterase inhibitor. *European Journal of Pharmacology*.

[48] Park K.-R., Yun H.-M., Quang T.-H. (2016). 4-Methoxydalbergione suppresses growth and induces apoptosis in human osteosarcoma cells in vitro and in vivo xenograft model through down-regulation of the JAK2/STAT3 pathway. *Oncotarget *.

[49] Jia M., Chen L., Xin H.-L. (2016). A friendly relationship between endophytic fungi and medicinal plants: A systematic review. *Frontiers in Microbiology*.

[50] Liu R.-X., Li L., Wang Q., Wang W., Bi K.-S., Guo D.-A. (2005). Simultaneous determination of nine flavonoids in Dalbergia odorifera by LC. *Chromatographia*.

[51] Liu R., Ye M., Guo H., Bi K., Guo D.-A. (2005). Liquid chromatography/electrospray ionization mass spectrometry for the characterization of twenty-three flavonoids in the extract of Dalbergia odorifera. *Rapid Communications in Mass Spectrometry*.

[52] Ham S. A., Kang E. S., Yoo T. (2015). Dalbergia odorifera Extract Ameliorates UVB-Induced Wrinkle Formation by Modulating Expression of Extracellular Matrix Proteins. *Drug Development Research*.

[53] Choi H. S., Park J., Hwang J. S. (2017). A Dalbergia odorifera extract improves the survival of endotoxemia model mice by inhibiting HMGB1 release. *BMC Complementary and Alternative Medicine*.

[54] Liu R., Sun J., Bi K., Guo D.-A. (2005). Identification and determination of major flavonoids in rat serum by HPLC-UV and HPLC-MS methods following oral administration of Dalbergia odorifera extract. *Journal of Chromatography B*.

[55] Zhao Y., Guo Z., Zhang X., Liang X., Zhang Y. (2010). Off-line 2-D RPLC/RPLC method for separation of components in Dalbergia odorifera T. Chen. *Journal of Separation Science*.

[56] Zhang D.-Y., Zu Y.-G., Fu Y.-J. (2011). Negative pressure cavitation extraction and antioxidant activity of biochanin A and genistein from the leaves of Dalbergia odorifera T. Chen. *Separation and Purification Technology*.

[57] Lianhe Z., Xing H., Zhengxing C. (2011). Antioxidant activities of seed extracts from *Dalbergia odorifera* T. Chen. *Afr. J. Biotechnol*.

[58] Lianhe Z., Xing H., Li W., Zhengxing C. (2012). Physicochemical properties, chemical composition and antioxidant activity of Dalbergia odorifera T. Chen seed oil. *Journal of the American Oil Chemists’ Society*.

[59] Amaral S., Mira L., Nogueira J. M. F., Silva A. P. D., Helena Florêncio M. (2009). Plant extracts with anti-inflammatory properties-A new approach for characterization of their bioactive compounds and establishment of structure-antioxidant activity relationships. *Bioorganic & Medicinal Chemistry*.

[60] Fakhrudin N., Waltenberger B., Cabaravdic M. (2014). Identification of plumericin as a potent new inhibitor of the NF-*κ*B pathway with anti-inflammatory activity *in vitro* and *in vivo*. *British Journal of Pharmacology*.

[61] Anh H. L. T., Kim D.-C., Ko W. (2017). Anti-inflammatory coumarins from Paramignya trimera. *Pharmaceutical Biology*.

[62] Scott J. P., Peters-Golden M. (2013). Antileukotriene agents for the treatment of lung disease. *American Journal of Respiratory and Critical Care Medicine*.

[63] Ward P. A., Sulavik M. C., Johnson K. J. (1984). Rat neutrophil activation and effects of lypoxygenase and cyclooxygenase inhibitors. *The American Journal of Pathology*.

[64] Chen C., Yang F.-Q., Zhang Q., Wang F.-Q., Hu Y.-J., Xia Z.-N. (2015). Natural products for antithrombosis. *Evidence-Based Complementary and Alternative Medicine*.

[65] Stitham V. P., Ying L., Hwa J. (2014). Cardiovascular pharmacogenetics of anti-thrombotic agents and non-steroidal anti-inflammatory drugs. *Current Molecular Medicine*.

[66] Assender J. W., Southgate K. M., Hallett M. B., Newby A. C. (1992). Inhibition of proliferation, but not of Ca^2+^ mobilization, by cyclic AMP and GMP in rabbit aortic smooth-muscle cells. *Biochemical Journal*.

[67] Smith V. B., Spina D., Page C. P. (2006). Phosphodiesterase inhibitors. *Bristish Journal of Pharmacology*.

[68] Nishida N., Yano H., Nishida T., Kamura T., Kojiro M. (2006). Angiogenesis in cancer. *Vascular Health and Risk Management*.

[69] Wang S., Zheng Z., Weng Y. (2004). Angiogenesis and anti-angiogenesis activity of Chinese medicinal herbal extracts. *Life Sciences*.

[70] Fan Z., Wang D., Yang J. (2017). Dalbergia odorifera extract promotes angiogenesis through upregulation of VEGFRs and PI3K/MAPK signaling pathways. *Journal of Ethnopharmacology*.

[71] Alon T., Hemo I., Itin A., Pe’er J., Stone J., Keshet E. (1995). Vascular endothelial growth factor acts as a survival factor for newly formed retinal vessels and has implications for retinopathy of prematurity. *Nature Medicine*.

[72] Jope R. S., Yuskaitis C. J., Beurel E. (2007). Glycogen synthase kinase-3 (GSK3): inflammation, diseases, and therapeutics. *Neurochemical Research*.

[73] Jope R. S., Johnson G. V. W. (2004). The glamour and gloom of glycogen synthase kinase-3. *Trends in Biochemical Sciences*.

[74] Liu P., Cheng H., Roberts T. M., Zhao J. J. (2009). Targeting the phosphoinositide 3-kinase pathway in cancer. *Nature Reviews Drug Discovery*.

